# New Terpenoids and Lignans from *Phyllanthus acidus* Fruits with Antioxidant Activity

**DOI:** 10.3390/foods14030452

**Published:** 2025-01-30

**Authors:** Ying Xin, Jia Xu, Na Li, Li-Ying Yang, Hong-Tao Zhu, Ying-Jun Zhang

**Affiliations:** 1 Key Laboratory of Phytochemistry and Natural Medicines, Kunming Institute of Botany, Chinese Academy of Sciences, Kunming 650201, China; xinying@mail.kib.ac.cn (Y.X.); 13430669492@163.com (J.X.); lina1@mail.kib.ac.cn (N.L.); 15087150616@163.com (L.-Y.Y.); zhuhongtao@mail.kib.ac.cn (H.-T.Z.); 2 Department of Pharmacy, Chongqing Three Gorges Medical College, Chongqing 404120, China; 3 University of Chinese Academy of Sciences, Beijing 100049, China

**Keywords:** antioxidant food, *Phyllanthus acidus* fruits, lignans, terpenoids

## Abstract

The fruits of *Phyllanthus acidus*, rich in various secondary metabolites and possessing significant antioxidant activity, have been consumed widely by many Southeast Asian people, including the Thai, Vietnamese, Burmese, Laotians, and Cambodians. An extensive investigation of the secondary metabolites of the fruits resulted in our obtaining 17 compounds, including four new compounds (**1**–**4**). The absolute configurations of **1**, **3**, and **4** were determined by comparing their experimental electronic circular dichroism (ECD) spectra with both reference data and computed ECD profiles. At a concentration of 40μM, terpenoids (**1** and **5**–**9**) showed no cytotoxic activity against five strains of human tumor cells and one of normal cells. Notably, the known lignan **13** and phenylpropanoid **15** showed obvious ABTS^+^ radical scavenging activities with IC_50_ values of 203.7 and 232.9 μM, which have a comparable impact to the positive control, Trolox (IC_50_ = 176.5 ± 2.0 μM). The results indicated that *P. acidus* fruits could be a promising sources of antioxidant food supplement.

## 1. Introduction

**Figure 1:**
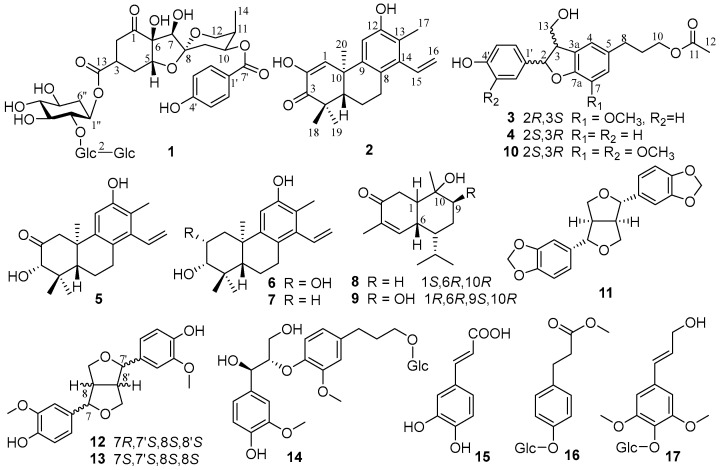
Compounds **1**–**17** isolated from the fruits of *Phyllanthus acidus*.

*Phyllanthus acidus* (Linn.) Skeels is primarily distributed in Thailand, Vietnam, Myanmar, Laos, and Cambodia. In recent decades, it has been cultivated in the Xishuangbanna and Yuanjiang regions of Yunnan Province, China. The fruits, featuring yellow-green peel and white juicy flesh that surrounds a pit of seeds, are sour, with a tart taste, and have been commercially packaged for sale in Thailand, Vietnam, Myanmar, Laos, and Cambodia. They are usually consumed with salt, but are eaten fresh as well. They have also been adapted into various cuisines and used to make syrups, pickles, and sweetened dried fruit or combined with other fruits to produce jams, across various geographic locales. Furthermore, the fruit juice is employed to produce beverages and vinegar [1]. Due to the potent antioxidant effects, the fruit could be used to protect cardiovascular and liver health when consumed regularly [2,3,4,5]. Up to now, studies on *P. acidus* were primarily interested in the chemical components in its leaves, roots, and stems, and resulted in the identification of a series of sesquiterpenoids, diterpenoids, triterpenoids, and flavonoids [6,7,8,9,10,11]. Among them, sesquiterpenoids exhibited possible anti-HBV effects, with IC_50_ ranges of 0.8 to 36 μM, targeting HBsAg and HBeAg. Diterpenoids and triterpenoids show potential for cytotoxic activities. This fruit, widely consumed in Southeast Asia, has rarely been studied for its chemical composition. Clarifying the chemical constituents of the fruit is vital for its further development and utilization. Our previous study of the fresh fruits resulted the separation of 13 flavonoids exhibiting potential *α*-glucosidase inhibitory activities [12,13]. Further chemical research on the fruits resulted in the isolation of four new (**1**–**4**) and 13 known compounds (Figure 1). The structures were clarified by extensive analysis of the NMR, HRESIMS, and ECD data. The phenolic compounds **3**–**4** and **10**–**17** were evaluated for their antioxidant activities. Meanwhile, the cytotoxic effects of terpenoids **1** and **5**–**9** were assessed on five human cancer cell lines (HL-60, A549, SMMC-7721, MDA-MB-231, and SW480) as well as on two human normal cell lines (BEAS-2B and L02).

## 2. Materials and Methods

### 2.1. General Procedure

UV data were collected by a Shimadzu UV-2401A spectrophotometer (Shimadzu, Kyoto, Japan). 1D and 2D NMR data were acquired using Bruker DRX-600 and -800 spectrometers (Bruker, Karlsruhe, Germany) in CD_3_OD working at 600 and 800 MHz for ^1^H, and 150 and 200 MHz for ^13^C, respectively, with tetramethylsilane (TMS) as internal standard. Chemical shifts (*δ*) are reported in ppm, with the TMS signal serving as the reference point. MS analyses were performed on Agilent 1290 UPLC/6540 Q-TOF mass spectrometer(Agilent Technologies Inc., Santa Clara, California, USA). Some materials used in column chromatography (CC) included Diaion HP20 (Mitsubishi Chemical Corporation, Tokyo, Japan), silica gel (200–300 mesh, Qingdao Marine Chemical, Inc., Qingdao, China), and RP-18 (40–60 μm, Merck, Darmstadt, Germany). A Waters 600-2487 instrument was used to purify the compounds.

### 2.2. Plant Material

The fruits were gathered from the Yuanjiang region, located at geographical coordinates 102° E and 23.6° N and an altitude of 327 m, in Yunnan Province of PRC, in June 2019. A total of 126 kg of fruit was collected from 50 trees. The plant material was authenticated by Dr. En-De Liu from the Kunming Institute of Botany (KIB), Chinese Academy of Sciences (CAS). A voucher specimen (Kib-18-05-22) was deposited in the KIB, CAS.

### 2.3. Extraction and Isolation

The entire extraction, separation, and identification experiment was carried out twice. The first round focused mainly on the higher polar part; however, due to the limited amount of sample available, the research on the less polar part was insufficient. Consequently, additional samples were collected for a second round of study, which focused specifically on the less polar part.

In the first round, after removing the kernels, the pulps (26 kg) were soaked in 50 L of 80% aqueous acetone. The extracts (1.6 kg) were extracted with ethyl acetate, resulting in an aqueous layer of 1.2 kg and an EtOAc layer of 50 g. The former was purified by Diaion HP-20 column chromatography (CC) (18 × 80 cm, MeOH/H_2_O 0:10, 3:7, 6:4, 8:2, and 10:0) to obtain five fractions, Fr. I-V.

Fr. III (10.0 g) was purified using RP-18 column (MeOH/H_2_O 1:0-0:1) and silica gel column (chloroform/methanol 100:1-1:1) to obtain nine sub-fractions Fr. III-1-III-9. Compound **15** (3.0 mg) was obtained by purifying Fr. III-6 (20 mg) via semi-preparative HPLC (15% MeCN/H_2_O, R*_t_* = 15.0 min, 3.0 mg), and **16** was similarly obtained (15% MeCN/H_2_O, R*_t_* = 24.0 min, 3.0 mg). Semi-preparative HPLC purification yielded compounds **17** (12% MeCN/H_2_O, R*_t_* = 11.4 min, 20.0 mg) from Fr. III-7 (30 mg) and **14** (15% MeCN/H_2_O, R*_t_* = 18.0 min, 6.0 mg) from Fr. III-8 (15 mg), respectively. Fr. IV (5.0 g) was sequentially subjected to RP-18 and silica gel CC to obtain the crystalline, compound **11** (22 mg), and then compound **1** was subjected to semi-preparative HPLC (13% MeCN/H_2_O, R*_t_* = 9.5 min, 5.0 mg).

The EtOAc fraction was separated by RP-18 CC (MeOH/H_2_O 1:9-10:0), yielding six fractions, Fr. VI-XI. Fr. IX (2.0 g) was applied to silica gel CC, followed with semi-preparative HPLC (15% MeCN/H_2_O) to obtain compound **13** (R*_t_* = 15.0 min, 3.0 mg). Fr. X (2.0 g) was subjected to a silica gel CC to acquire seven sub-fractions, Fr. X-1-X-7. Compound **2** was obtained by purifying Fr. X-2 via semi-preparative HPLC (60% MeCN/H_2_O, R*_t_* = 14.4 min, 3 mg), and **5** (55% MeCN/H_2_O, R*_t_* = 22.0 min, 1.5 mg), **7** (55% MeCN/H_2_O, R*_t_* = 23.2 min, 1.0 mg), and **8** (42% MeCN/H_2_O, R*_t_* = 14.0 min, 2.0 mg) were similarly obtained. Compound **6** was isolated from Fr. X-3 by semi-preparative HPLC purification (55% MeCN/H_2_O, R*_t_* = 15.4 min, 5.0 mg), and **9** was similarly obtained (48% MeCN/H_2_O, R*_t_* = 18.5 min, 2.0 mg).

In the second round, 100 kg of fresh fruits with kernels (*P. acidus*) was crushed and then extracted using 200 L of aqueous acetone (80%) at ambient temperature. The yielded extract (7.0 kg) was partitioned between water and ethyl acetate to obtain the aqueous (6.1 kg) and EtOAc (630 g) extracts. The later was applied to a silica gel CC (chloroform/methanol 100:1, 50:1, 20:1, and 10:1) to obtain four fractions, Fr. I-IV. Fr. II (20 g) was separated by CC over RP-18 (MeOH/H_2_O 1:0-0:1) and silica gel (PE/EtOAc 100:1-10:1), subsequently purified, and then furnished compounds **3** (38% MeCN/H_2_O, R*_t_* = 15.2 min, 1.7 mg), **4** (38% MeCN/H_2_O, R*_t_* = 18.0 min, 1.3 mg), and **10** (38% MeCN/H_2_O, R*_t_* = 16.0 min, 3.5 mg). Fr. III (20 g) was purified using silica gel (PE/EtOAc 100:1-10:1) and preparative HPLC to obtain compounds **12** (35% MeCN/H_2_O, R*_t_* = 9.6 min, 1.2 mg) and **13** (35% MeCN/H_2_O, R*_t_* = 11.1 min, 1.3 mg).

#### 2.3.1. Phylanthacidoid V (**1**)

Light-yellow amorphous powder: ${\left[\alpha \right]}_{D}^{25}$ − 14.63 (*c* 0.19, MeOH). UV (MeOH) *λ*_max_ (log *ε*) 227.0 (0.17) nm, 260.0 (0.52) nm; NMR (600 MHz, methanol-*d*_4_) see Table 1; and HRESIMS *m*/*z* 905.2932 [M − H]^−^ (calcd for C_39_H_53_O_24_, 905.2933).

#### 2.3.2. Phyllaciduloid H (**2**)

Light-yellow amorphous powder: NMR (600 MHz, methanol-*d*_4_) see Table 1 and HRESIMS *m*/*z* 311.1660 [M − H]^−^ (calcd for C_20_H_23_O_3_, 311.1653).

#### 2.3.3. 3-[(2R,3S)-2-(4-Hydroxyphenyl)-3-(hydroxymethyl)-7-methoxy-2,3-dihydro-1-benzofuran-5-yl]propyl Acetate (**3**)

Yellowish oil: ${\left[\alpha \right]}_{D}^{25}$ +15.58 (*c* 0.05, MeOH). UV (MeOH) *λ*_max_ (log *ε*) 196 (0.66) nm, 229 (0.29) nm; 281.5 (0.05) nm; NMR (600 MHz, methanol-*d*_4_) see Table 2; and HRESIMS *m*/*z* 371.1495 [M − H]^−^, (calcd for C_21_H_23_O_6_, 371.1500).

#### 2.3.4. 3-[(2S,3R)-2-(4-Hydroxyphenyl)-3-(hydroxymethyl)-2,3-dihydro-1-benzo-furan-5-yl]pro-pyl Acetate (**4**)

Yellowish oil: ${\left[\alpha \right]}_{D}^{25}$ +4.00 (*c* 0.12, MeOH). UV (MeOH) *λ*_max_ (log *ε*) 196.5 (0.70) nm, 226.5 (0.17) nm, 283.5 (0.05) nm; NMR (600 MHz, methanol-*d*_4_) see Table 2; and HRESIMS *m*/*z* 341.1388 [M − H]^−^ (calcd for C_20_H_21_O_5_, 341.1394).

### 2.4. Antioxidant Assay

The ABTS^+^ radical scavenging test was conducted as reported previously, and was slightly modified [14]. Specifically, the solvent was replaced with DMSO due to the high volatility of 80% methanol. Trolox was used as positive control. The scavenging activities of phenolic compounds **3**, **4**, and **10**–**17** were calculated based on the percentage of ABTS^+^ radical scavenged using the equation:

Inhibition rate (%) = (A _blank_ − A _sample_)/A _blank_

IC_50_ values were calculated according to the Reed and Muench method [15].

### 2.5. Cytotoxicity Assay

The cytotoxic activities of terpenoids (**1** and **5**–**9**) were assessed. Five human tumor cell lines (HL-60, A549, SMCC-7721, MDA-MB-231, and SW480) and two human normal cell lines (BEAS-2B and L02) were used in cytotoxic assay, and were sourced from ATCC (Manassas, VA, USA). Cells were grown in RMPI-1640 or DMEM medium (Biological Industries, Kibbutz Beit-Haemek, Israel), with the addition of 10% fetal bovine serum. In short, cells were placed into each well of a 96-well culture plate. Following a 12–24 h incubation period, the terpenoids (40 μM) were introduced, with DDP and Taxol serving as positive controls. After 48 h of incubation at 37 °C, cells evaluated using the MTS assay. Amounts of 20 μL of MTS solution and 100 μL of culture medium were added to each well; 100 μL of supernatant from the suspended cells was discarded, and 20 μL of MTS solution was added to each well; three blank duplicate wells were set up (containing a mixture of 20 μL of MTS solution and 100 μL of culture medium); and the incubation was continued for 2 to 4 h to allow the reaction to proceed fully before the optical absorbance values were measured. A wavelength of 492 nm was selected, and the Multiskan FC multifunctional plate reader was used to measure the absorbance values of each well. The results are recorded and, after data processing, the inhibition rate curves for the five cell lines are plotted with the compound number on the x-axis and the cell inhibition rate on the *y*-axis.

## 3. Results and Discussion

### 3.1. Identification of Compounds ***1**–**17***

The extract of *P. acidus* fruits was partitioned between H_2_O and EtOAc. Further various column chromatography, followed with semi-preparative HPLC, yielded 17 compounds, including four new ones (**1**–**4**). The known compounds were determined to be three cleistanthane diterpenes, aspidoptoid D (**5**) [16], cleistanthol (**6**), and spruceanol (**7**) [17]; two monoterpenes, 10*α*-hydroxyamorph-4-en-3-one (**8**) [18] and hypocreaterpene B (**9**) [19]; one benzofuran lignan, 3-[(2*S*,3*R*)-2-(4-hydroxy-3-methoxyphenyl)-3-(hydroxymethyl)-7-methoxy-2,3-dihydro-1-benzofuran-5-yl]propyl acetate (**10**) [20]; three bis-tetrahydrofuran lignans, sesamin (**11**) [21], (+) epipinoresinol (**12**) [22], and (-)-pinoresinol (**13**) [23]; one neolignan, 4,7,9-trihydroxy-3,3′-dimethoxy-8-*O*-4′-neolignan-9′-*O*-*β*-D-glucopyranoside (**14**) [24]; and three phenylpro-panoids, *trans*-caffeic acid (**15**) [25], methyl-3-(4-*O*-*β*-D-glucopyranosylphenyl)propio-nate (**16**) [26], and syringin (**17**) [27], respectively, by matching their spectroscopic information with previously published data.

**Figure 2:**
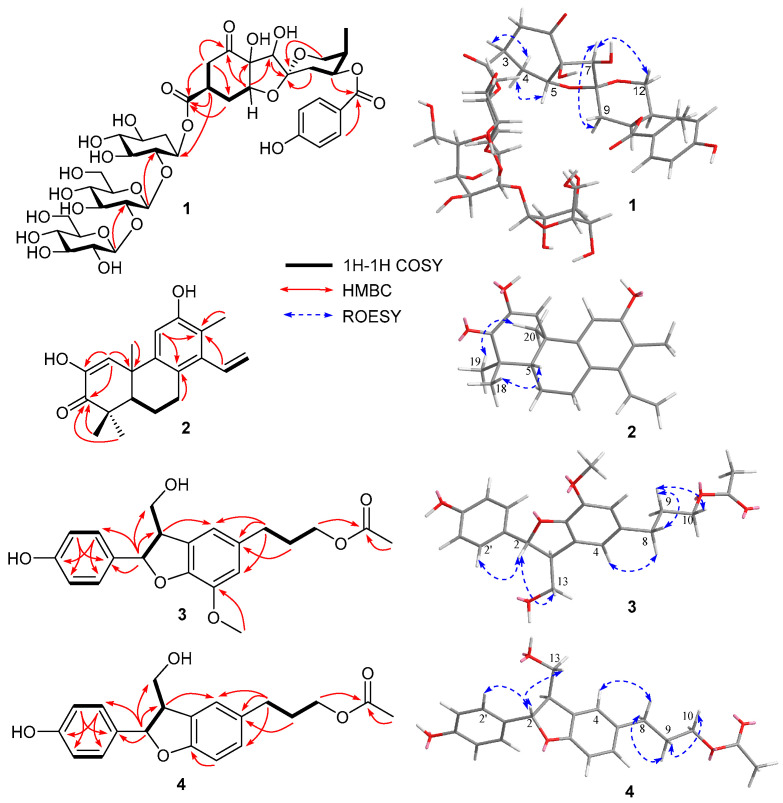
Key 2D correlations in compounds **1–4**.

**Figure 3:**
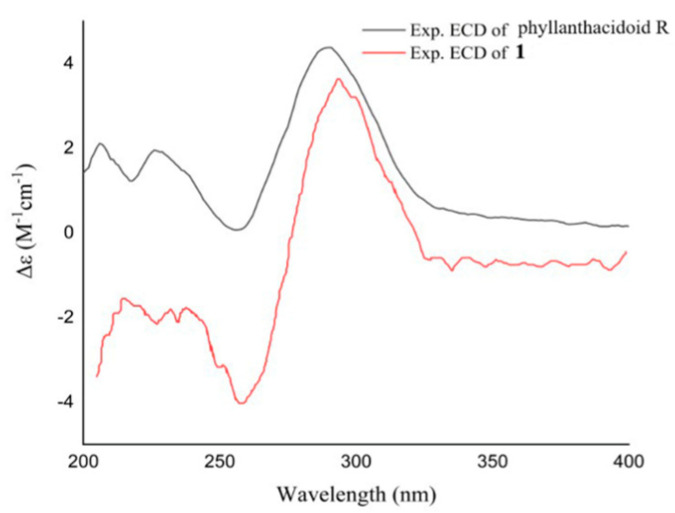
Experimental ECD curves of **1** and phyllanthacidoid R.

Phyllanthacidoid V (**1**) was yielded as a light-yellow amorphous powder. Its molecular formula, C_39_H_54_O_24_, was determined by the HRESIMS (*m*/*z* 905.2932 [M − H]^−^) and ^13^C NMR spectrum, requiring 13 degrees of unsaturation. The ^13^C NMR and DEPT data revealed the existence of 39 carbon signals, consisting of four quaternary carbons with a ketone (*δ*_C_ 211.8), a carboxyl (*δ*_C_ 175.5), a ketal (*δ*_C_ 109.9), and an oxygen-bearing one (*δ*_C_ 85.5). Moreover, five methine resonances with three oxygen-bearing ones (*δ*_C_ 84.7, 87.5, and 71.6), four oxygen-bearing methylenes (*δ*_C_ 65.7), and one methyl (*δ*_C_ 10.8) were observed, in addition to 18 signals arising from a cyclohexan (*δ*_C_ 71.1, 85.4, 75.7, 77.4, 70.0, and 35.8) and two hexosyl moieties (*δ*_C_ 104.2, 85.3, 77.6, 70.7, 77.6, 62.4, 106.3, 76.5, 79.0, 71.6, 78.9, and 62.8), and seven signals from a *p*-hydroxybenzoyl unit (*δ*_C_ 132.9×2, 116.4×2, 122.5, 163.8, and 167.6) (Table 1). The data mentioned earlier exhibited a strong resemblance to those of phyllanthacidoid R [10], reported to be from the same plant, suggesting that **1** was a norbisabolane sesquiterpene. The only difference lies in the additional sugar signal (*δ*_C_ 106.3, 76.5, 79.0, 71.6, 78.9, and 62.8) in **1,** compared to phyllanthacidoid R. The cyclohexan and two hexosyl moieties were determined to be from one pentaoxy cyclohexane unit and two glucosyl units, which was consistent with phyllanthanidoid H [10]. An in-depth analysis of the HMBC correlations of H-1′′′ (*δ*_H_ 4.76) with C-2′′ (*δ*_C_ 85.4), H-1′′′′ (*δ*_H_ 4.61) with C-2′′′ (*δ*_C_ 85.3), and H-3 (*δ*_H_ 3.43) with C-1′′ (*δ*_C_ 71.1) further revealed that a pentaoxy cyclohexane moiety was attached to position C-13 via an ester linkage (Figure 2). Moreover, the large coupling constants of *J*_H-1′′′, H-2′′′_ (7.8 Hz) and *J*_H-1′′′′, H-2′′′′_ (7.9 Hz) indicated the configurations of glucose were both *β*-D. The relative configuration of **1** was confirmed by comparison with known compounds, coupling constant analysis and ROESY correlations. The coupling constant of H-3 (ddd, *J* = 3.6, 6.6, and 11.4 Hz) and low coupling constant of H-5 (t, *J* = 4.0 Hz) indicated the axial orientation of H-3 and equatorial orientation of H-5. Furthermore, the ROESY correlations of H-3 (*δ*_H_ 3.43) with H-4eq (*δ*_H_ 2.45), and of H-5 (*δ*_H_ 4.29) with H-4ax (*δ*_H_ 1.99) suggested that H-3 and H-4eq were in the same orientation (*α*-orientation) and that H-5 and H-4ax were on the reverse side (*β*-orientation). Ring C possessed the identical relative configuration as phyllanthacidoid R. The ROESY correlation between H-7 and H-9ax permitted H-7 to have an *α*-orientation. In addition, the ROESY cross-peak of H-7 with H-12 confirmed that both H-7 and C-12 were *α*-oriented. Based on the aforementioned evidence, the relative configuration of **1** was identical to that of phyllanthacidoid R [10]. Compound **1** generated a Cotton effect similar to that of phyllanthacidoid R (Figure 3). Hence, the absolute configuration of compound **1** was established as 3*S*,5*R*,6*S*,7*R*,8*R*,10*S*, and 11*R*.

Phyllaciduloid H (**2**) was isolated as a light-yellow amorphous powder. A molecular formula of C_20_H_23_O_3_ was deduced from the (-)-HRESIMS at *m*/*z* 311.1660 [M − H]^−^ (calcd for C_20_H_23_O_3_, 311.1653), with nine degrees of unsaturation. The ^13^C NMR and DEPT spectra (Table 1) showed 20 carbon signals, including four methyls (*δ*_C_ 29.6, 27.3, 22.0, and 13.3), three methylenes (*δ*_C_ 119.8, 29.4, and 20.4) with one olefinic, four methines (*δ*_C_ 137.1, 128.5, 110.2, and 49.0) with three olefinic, and nine quaternary (*δ*_C_ 201.9, 154.6, 146.6, 144.0, 141.0, 124.6, 121.5, 45.4, and 40.0) with one ketone (*δ*_C_ 201.9), six olefinic (*δ*_C_ 154.6, 146.6, 144.0, 141.0, 124.6, and 121.5), and two aliphatic carbons (*δ*_C_ 45.4 and 40.0). The above features suggested **2** to be a cleistanthane-type diterpenoid with very similar structure to compound **5**. The only difference was that **2** possessed two additional double-bond signals, compared with **5**. Moreover, in the HMBC spectrum of **2**, correlations of H-1 (*δ*_H_ 6.75) with C-3 (*δ*_C_ 201.9), H-18 (*δ*_H_ 1.22)/H-19 (*δ*_H_ 1.17) with C-3 (*δ*_C_ 201.9), and H-1 with C-2 (*δ*_C_ 146.6) confirmed the connection of C-1/C-2/C-3. The ^13^C chemical shift of C-2 (*δ*_C_ 146.6) suggested that it was an oxygen-bearing olefinic carbon. This deduction was verified by the HMBC correlations of H-1 (*δ*_H_ 6.75) with C-2 (*δ*_C_ 146.6) and C-10 (*δ*_C_ 40.0). Thus, the planar structure of **2** was finally determined. The ROESY correlations (Figure 2) of H-19/H-20 and H-5/H-18 corroborated that H-18 and H-5 exhibited *β*-orientation, while H-19 and H-20 exhibited *α*-orientation. On biogenetic grounds, compound **2** was confirmed as an *ent*-cleistanthane and named as phyllaciduloid H.

**Figure 4:**
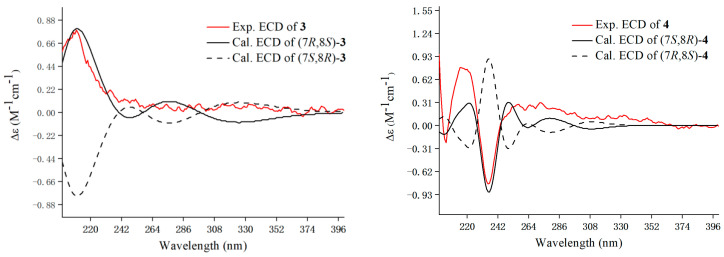
Experimental and calculated ECD curves of **3** and **4**.

Compounds **3** was yielded as a yellowish oil. The molecular formula (C_21_H_24_O_6_) was assigned based on the HRESIMS (*m*/*z* 371.1495 [M − H]^−^, calcd for C_21_H_23_O_6_, 371.1500), indicating 10 unsaturation degrees. The ^13^C NMR data of **3** revealed the existence of 21 carbon signals that can be attributed to a lignan skeleton consisting of two benzene rings (*δ*_C_ 114.2–158.6), one aliphatic (*δ*_C_ 55.5) and one oxygen-bearing (*δ*_C_ 89.0) methine, and two aliphatic (*δ*_C_ 33.1, 31.9) and two oxygen-bearing (*δ*_C_ 65.2 × 2) methylenes (Table 2). In addition, the remaining carbons included a methoxy group (*δ*_C_ 56.9) and an acetyl (*δ*_C_ 173.3, 21.0). In the ^1^H NMR spectrum, H-4, H-6 (*δ*_H_ 6.72) arising from a benzene system, and H-2′/6′, H-3′/5′ (*δ*_H_ 7.20, 6.75, each 2H, d, *J* = 8.6 Hz) corresponding to a 1,4-disubstituted benzene, were presented. A thorough analysis of NMR data between **3** and **10** denoted that their structures were quite similar, with the exception that compound **3** has no methoxy signal at C-5′. The inference was further verified by the HMBC and ^1^H-^1^H COSY correlations. The cross peaks of H-2′/6′ with H-3′/5′, H-3 with H-2/H-13, and H-9 with H-8/H-10 were observed in a COSY spectrum. Meanwhile, the HMBC correlations from H-3 (*δ*_H_ 3.45) to C-4 (*δ*_C_ 118.1), H-8 (*δ*_H_ 2.65) to C-5 (*δ*_C_ 136.2)/C-4 (*δ*_C_ 118.1)/C-6 (*δ*_C_ 114.2), H-9 (*δ*_H_ 1.94) to C-5, H-10 (*δ*_H_ 4.07)/H-12 (*δ*_H_ 2.04) to C-11 (*δ*_C_ 173.3), and -OCH_3_ (*δ*_H_ 3.85) to C-7 (*δ*_C_ 145.4) illustrated the planar structure of compound **3**. The strong cross peak at H-2/H-13 in the ROESY analysis indicated a *trans* orientation of H-2 and H-3 (Figure 2). Further ECD calculations were performed to determine the absolute configuration of **3**. The calculated ECD spectrum of (2*R*,3*S*) matched well with the experimental one (Figure 4). Thus, compound **3** was finally named as 3-[(2*R*,3*S*)-2-(4-hydroxyphenyl)-3-(hydroxymethyl)-7-methoxy-2,3-dihydro-1-benzofuran-5-yl]propyl acetate.

The molecular formula of **4** was assigned to be C_20_H_22_O_5_, according to its HRESIMS data. The 1D NMR spectra, showing 20 carbon signals, indicated that **4** shares the same structural skeleton as that of **3**. The only difference between **3** and **4** is the absence of a methoxy signal in **4**. Instead of the 1,3,4,5-tetrasubsituted benzene ring present in **3**, **4** features an ABX coupled benzene ring with signals at *δ*_H_ 7.10 (1H, d, *J* = 1.9 Hz), 7.00 (1H, d, *J* = 1.9, 8.1 Hz), and 6.71 (1H, d, *J* = 8.1 Hz). This was supported by the HMBC correlation from H-7 (*δ*_H_ 6.71) to C-7a (*δ*_C_ 159.7). The relative configurations of **4** were verified as being identical to those of **3** based on the comparable ROESY correlations (Figure 2). The absolute configuration of **4** was verified as shown, by comparing the ECD curve with that of our calculation (Figure 4). The structure of 3-[(2*S*,3*R*)-2-(4-hydroxyphenyl)-3-(hydroxymethyl)-2,3-dihydro-1-benzofuran-5-yl] propyl acetate (**4**) was, thus, assigned as shown.

### 3.2. Antioxidant Activity

Most of the isolated lignans and phenylpropanoids (**3**, **4**, and **10**–**17**) were evaluated for their ABTS^+^ radical scavenging abilities, with Trolox as the positive control. Compounds **13** and **15** exhibited notable antioxidant activities against ABTS^+^ radicals (IC_50_ = 203.7–232.9 μM), and had equivalent activities to the positive control Trolox (IC_50_ = 176.5 μM) (Table 3). In addition, **10**, **12**, and **14** showed moderate activities with IC_50_ values 348.4 to 387.4 μM. The antioxidant activities of (−)-pinoresinol (**13**) from *Liriope muscari* and *Senecio scandens* were comparable to that of Vitamin C [28,29]. In addition, *trans*-caffeic acid (**15**) from *Cordia sinensis* demonstrates better DPPH radical scavenging activity than BHA (Butyl hydroxy anisd) [30]. (+)-Epipinoresinol (**12**) from *Forsythia suspensa* also shows similar activity [31]. In summary, these research findings are in agreement with previously mentioned results.

Compounds **10** and **12** and **13**–**15,** with more unsubstituted phenolic hydroxyl or carboxyl groups in their molecular structure, showed moderate or comparable free radical scavenging activities when compared to the positive control (Trolox). Thus, the number of unsubstituted phenolic hydroxyl and carboxyl groups directly affects antioxidant activity. The more phenolic hydroxyl and carboxyl groups there are, the stronger the antioxidant capacity. These compounds may exert antioxidant effects through the SET (single-electron transfer) mechanism. Initially, the carboxyl groups and phenolic hydroxyl groups or their ionic forms of the compounds can serve as electron donors, transferring electrons to the ABTS^+^ radical to form ABTS^−^. Subsequently, ABTS^−^ further reacts in the solution, leading to a decrease in absorbance. After losing electrons, carboxylic acids and phenolic hydroxyl groups or their ionic forms may form radical intermediates. These intermediates can further participate in other radical reactions, thereby enhancing the antioxidant effect.

### 3.3. Cytotoxic Activity

The cytotoxic effects of terpenoids **1** and **5**–**9** were assessed against five human tumor (HL-60, A549, SMMC-7721, MDA-MB-231, and SW480) and two human normal (BEAS-2B and L02) cell lines. However, all of them showed no significant cytotoxic activities at concentrations up to 40 μM. By referring to the initial screening concentrations of compounds with similar structures in the literature, a concentration of 40 μM was determined [11]. Aspidoptoid D (**5**) from *Aspidopterys obcordate* likewise exhibited no significant cytotoxic activity against five tumor cell lines at a concentration of 40 μM [18]. Additionally, cleistanthol (**6**) isolated from *Phyllanthus flexuosus* displayed weak cytotoxic activity against ECA109 [32]. Furthermore, cleistanthol (**6**) and spruceanol (**7**), both derived from *Phyllanthus acidus*, showed weaker cytotoxic activity against A549, HONE, and Hela cells [11]. Therefore, the results from the literature are generally consistent with the conclusions of this study.

## 4. Conclusions

The fruit of *P. acidus* is widely consumed in Southeast Asia. However, its chemical composition has rarely been studied. In the present study, four new compounds including one norbisabolane sesquiterpenoid glycoside (**1**), one cleistanthane diterpenoid (**2**), and two benzofuran lignans (**3**, **4**), along with 13 known compounds (**5**–**17**), were isolated and identified from the fruits of *P. acidus* for the first time. The isolated phenolic compounds (**10** and **12** and **13**–**15**) exhibited certain antioxidant activity, while terpenoids (**1** and **5**–**9**) showed no cytotoxicity at a concentration of 40 μM, indicating that the fruit can be served as an antioxidant food, and is safe and non-toxic. Hence, phenylpropanoids and lignans are the chemical basis for the antioxidant properties of the fruits. The result provides a scientific basis for developing *P. acidus* fruits as an ideal food supplement with antioxidant properties.

## Figures and Tables

**Table 1:** ^1^H (600 MHz) and ^13^C (150 MHz) data for compounds **1** and **2** in CD_3_OD (*δ* in ppm, *J* in Hz).

1					2			
No.	*δ*_C_	*δ*_H_	No.	*δ*_C_	*δ*_H_	No.	*δ*_C_	*δ*_H_
1	211.8		7′	167.6		1	128.5	6.75 (s)
2	42.1	2.58 (dd, 9.3, 18.4)	1′′	71.1	4.83 *^d^*	2	146.6	
		2.73 (dd, 6.8, 18.4)	2′′	85.4	3.26 *^c^*	3	201.9	
3	36.9	3.43 (ddd, 3.6, 6.6, 11.4)	3′′	75.7	3.43 *^c^*	4	45.4	
4	31.5	1.99 *^a^*	4′′	77.4	3.34 *^c^*	5	49.0	1.28 (m)
		2.45 (brd, 14.7)	5′′	70.0	3.48 (m)	6	20.4	1.87 (m)
5	84.7	4.29 (t, 4.0)	6′′	35.8	1.54 (q, 12.2)	7	29.4	2.73 (m)
6	85.5				2.13 *^a^*	8	124.6	
7	87.5	3.93 *^b^*	1′′′	104.2	4.76 (d, 7.9)	9	144.0	
8	109.9		2′′′	85.3	3.28 *^c^*	10	40.0	
9	30.7	2.01 *^a^*	3′′′	77.6	3.57 *^c^*	11	110.2	6.79 (s)
		2.13 *^a^*	4′′′	70.7	3.33 *^c^*	12	154.6	
10	71.6	5.51 (dt, 5.0, 11.0)	5′′′	77.6	2.14 (m)	13	121.5	
11	33.7	2.23 (m)	6′′′	62.4	3.40 (m)	14	141.0	
12	65.7	3.58 *^c^*			3.56 (m)	15	137.1	6.60 (dd, 18.0, 12.0)
		4.13 (dd, 2.7, 11.9)	1′′′′	106.3	4.61 (d, 7.8)	16	119.8	5.11 (dd, 11.4, 1.8)
13	175.5		2′′′′	76.5	3.33 *^c^*			5.51 (dd, 11.4, 1.8)
14	10.8	1.15 (3H, d, 7.0)	3′′′′	79.0	3.40 *^c^*	17	13.3	2.11 (s)
1′	122.5		4′′′′	71.6	3.38 *^c^*	18	27.3	1.22 (s)
2′/6′	132.9	7.89 (d, 8.6)	5′′′′	78.9	3.39 *^c^*	19	22.0	1.17 (s)
3′/5′	116.4	6.84 (d, 8.6)	6′′′′	62.8	3.71 *^c^*	20	29.6	1.37 (s)
4′	163.8				3.91 (m)			

*^a, b, c, d^* Signals were overlapped with each other or by solvents.

**Table 2:** ^1^H NMR (600 MHz) and^13^C NMR data for **3** and **4** in CD_3_OD (*δ* in ppm, *J* in Hz).

No.	3		4	
	*δ*_C_	*δ*_H_	*δ*_C_	*δ*_H_
2	89.0	5.49 (d, 6.1)	88.5	5.44 (d, 6.0)
3	55.5	3.45 (m)	55.1	3.43 (m)
3a	130.2		129.2	
4	118.1	6.72 (brs)	126.0	7.10 (d, 1.9)
5	136.2		135.0	
6	114.2	6.72 (brs)	129.9	7.00 (d, 1.9, 8.1)
7	145.4		110.0	6.71 (d, 8.1)
7a	147.9		159.7	
8	33.1	2.65 (t, 7.2)	32.8	2.64 (t, 7.6)
9	31.9	1.94 (m)	32.0	1.92 (m)
10	65.2	4.07 (t, 6.2)	65.2	4.06 (t, 6.5)
11	173.3		173.2	
12	21.0	2.04 (s)	21.0	2.03 (s)
13	65.2	3.74 (dd, 5.5, 10.9)	65.3	3.75 (dd, 7.3, 11.0)
		3.81 (dd, 7.1, 11.1)		3.81 (dd, 5.5, 11.0)
7-OCH_3_	56.9	3.85 (s)		
1′	134.3		134.6	
2′	128.5	7.20 (d, 8.5)	128.4	7.18 (d, 8.6)
3′	116.3	6.75 (d, 8.5)	116.4	6.75 (d, 8.6)
4′	158.6		158.5	
5′	116.3	6.75 (d, 8.5)	116.4	6.75 (d, 8.6)
6′	128.5	7.20 (d, 8.5)	128.4	7.18 (d, 8.6)

**Table 3:** Inhibitory activities of compounds **3**–**4**, **10**, and **12**–**15** in ABTS^+^ inhibition activities assay.

Compd.	Concentration (μM)	Inhibition Rate (%)	IC_50_ (μM)
Trolox	250	57.26 ± 1.38	176.5 ± 2.1
**3**	250	31.16 ± 0.38	-
**4**	250	23.71 ± 0.37	-
**10**	250	46.46 ± 0.83	348.4 ± 12.2
**12**	250	49.51 ± 1.60	387.4 ± 8.0
**13**	250	60.12 ± 0.19	203.7 ± 4.7
**14**	250	39.02 ± 1.35	361.8 ± 8.4
**15**	250	49.40 ± 0.72	232.9 ± 1.9

## Data Availability

The original contributions presented in this study are included in the article/Supplementary Materials. Further inquiries can be directed to the corresponding author.

## Supplementary Materials

The following supporting information can be downloaded at: https://www.mdpi.com/article/10.3390/foods14030452/s1. Figure S1. ^1^H NMR spectrum of compound **1**. Figure S2. ^13^C NMR spectrum of compound **1**. Figure S3. HSQC spectrum of compound **1**. Figure S4. HMBC spectrum of compound **1**. Figure S5. ^1^H-^1^H COSY spectrum of compound **1**. Figure S6. ROESY spectrum of compound **1**. Figure S7. HRESIMS spectrum of compound **1**. Figure S8. CD and UV spectra of compound **1**. Figure S9. ^1^H NMR spectrum of compound **2**. Figure S10. ^13^C NMR spectrum of compound **2**. Figure S11. HSQC spectrum of compound **2**. Figure S12. HMBC spectrum of compound **2**. Figure S13. ^1^H-^1^H COSY spectrum of compound **2**. Figure S14. ROESY spectrum of compound **2**. Figure S15. Negative ESIMS spectrum of compound **2**. Figure S16. HRESIMS spectrum of compound **2**. Figure S17. 1H NMR spectrum of compound **3**. Figure S18. 13C NMR spectrum of compound **3**. Figure S19. HSQC spectrum of compound **3**. Figure S20. HMBC spectrum of compound **3**. Figure S21. ^1^H-^1^H COSY spectrum of compound **3**. Figure S22. ROESY spectrum of compound **3**. Figure S23. HRESIMS spectrum of compound **3**. Figure S24. CD and UV spectra of compound **3**. Figure S25. ^1^H NMR spectrum of compound **4**. Figure S26. ^13^C NMR spectrum of compound **4**. Figure S27. HSQC spectrum of compound **4**. Figure S28. HMBC spectrum of compound **4**. Figure S29. ^1^H-^1^H COSY spectrum of compound **4**. Figure S30. ROESY spectrum of compound **4**. Figure S31. HRESIMS spectrum of compound **4**. Figure S32. CD and UV spectra of compound **4**. Figure S33. Flowchart of extraction and isolation. Table S1. Inhibitory activities of compounds **3**–**4**, **10**, **12**–**15** in ABTS^+^ inhibition activities assay. Table S2. Gibbs free energies and equilibrium populations of low-energy conformers of 3 *R*/*S*. Table S3. Cartesian coordinates for the low-energy reoptimized random reseach conformers of 3 *R*/*S* at B3LYP-D3(BJ)/6-31G* level of theory in methanol. Table S4. Gibbs free energies and equilibrium populations of low-energy conformers of 4 *R*/*S*. Table S5. Cartesian coordinates for the low-energy reoptimized random reseach conformers of 4 *R*/*S* at B3LYP-D3(BJ)/6-31G* level of theory in methanol. Refs. [33,34] are cited in Supplementary Materials.
