# Supplementary material for: New Terpenoids and Lignans from Phyllanthus acidus Fruits with Antioxidant Activity
Source: Foods. 2025 Jan 30;14(3):452. doi: 10.3390/foods14030452 (PMC11816990; doi:10.3390/foods14030452)
Supplement: Supplementary file 1 [file foods-14-00452-s001.zip › foods-3404621-supplementary.pdf]

Supporting Information

New Terpenoids and Lignans from *Phyllanthus acidus* Fruits with Antioxidant Activity

Ying Xin [1,2,3,†], Jia Xu [1,†], Na Li [1], Li-Ying Yang [1], Hong-Tao Zhu [1] and Ying-Jun Zhang [1,*]

[1] Key Laboratory of Phytochemistry and Natural Medicines, Kunming Institute of Botany, Chinese Academy of Sciences, Kunming 650201, China
[2] Department of Pharmacy, Chongqing Three Gorges Medical College, Chongqing 404120, China
[3] University of Chinese Academy of Sciences, Beijing 100049, China
* Correspondence: zhangyj@mail.kib.ac.cn
† These authors contributed equally to this work.

Contents of Supporting Information

No.	Contents
1.	Figure S1. ^{1}H NMR spectrum of compound **1**
2.	Figure S2. ^{13}C NMR spectrum of compound **1**
3.	Figure S3. HSQC spectrum of compound **1**
4.	Figure S4. HMBC spectrum of compound **1**
5.	Figure S5. ^{1}H-^{1}H COSY spectrum of compound **1**
6.	Figure S6. ROESY spectrum of compound **1**
7.	Figure S7. HRESIMS spectrum of compound **1**
8.	Figure S8. CD and UV spectra of compound **1**
9.	Figure S9. ^{1}H NMR spectrum of compound **2**
10.	Figure S10. ^{13}C NMR spectrum of compound **2**
11.	Figure S11. HSQC spectrum of compound **2**
12.	Figure S12. HMBC spectrum of compound **2**
13.	Figure S13. ^{1}H-^{1}H COSY spectrum of compound **2**
14.	Figure S14. ROESY spectrum of compound **2**
15.	Figure S15. Negative ESIMS spectrum of compound **2**
16.	Figure S16. HRESIMS spectrum of compound **2**
17.	Figure S17. ^{1}H NMR spectrum of compound **3**
18.	Figure S18. ^{13}C NMR spectrum of compound **3**

19. Figure S19. HSQC spectrum of compound **3**

20. Figure S20. HMBC spectrum of compound **3**

21. Figure S21. ^{1}H-^{1}H COSY spectrum of compound **3**

22. Figure S22. ROESY spectrum of compound **3**

23. Figure S23. HRESIMS spectrum of compound **3**

24. Figure S24. CD and UV spectra of compound **3**

25. Figure S25. ^{1}H NMR spectrum of compound **4**

26. Figure S26. ^{13}C NMR spectrum of compound **4**

27. Figure S27. HSQC spectrum of compound **4**

28. Figure S28. HMBC spectrum of compound **4**

29. Figure S29. ^{1}H-^{1}H COSY spectrum of compound **4**

30. Figure S30. ROESY spectrum of compound **4**

31. Figure S31. HRESIMS spectrum of compound **4**

32. Figure S32. CD and UV spectra of compound **4**

33. Figure S33. Flowchart of extraction and isolation

34. Table S1. Inhibitory activities of compounds **3-4**, **10**, **12-15** in ABTS^{+} inhibition activities assay

35. Table S2. Gibbs free energies and equilibrium populations of low-energy conformers of **3** *R/S*.

36. Table S3. Cartesian coordinates for the low-energy reoptimized random reseach conformers of **3** *R/S* at B3LYP-D3(BJ)/6-31G* level of theory in methanol.

37. Table S4. Gibbs free energies and equilibrium populations of low-energy conformers of **4** *R/S*.

38. Table S5. Cartesian coordinates for the low-energy reoptimized random reseach conformers of **4** *R/S* at B3LYP-D3(BJ)/6-31G* level of theory in methanol.

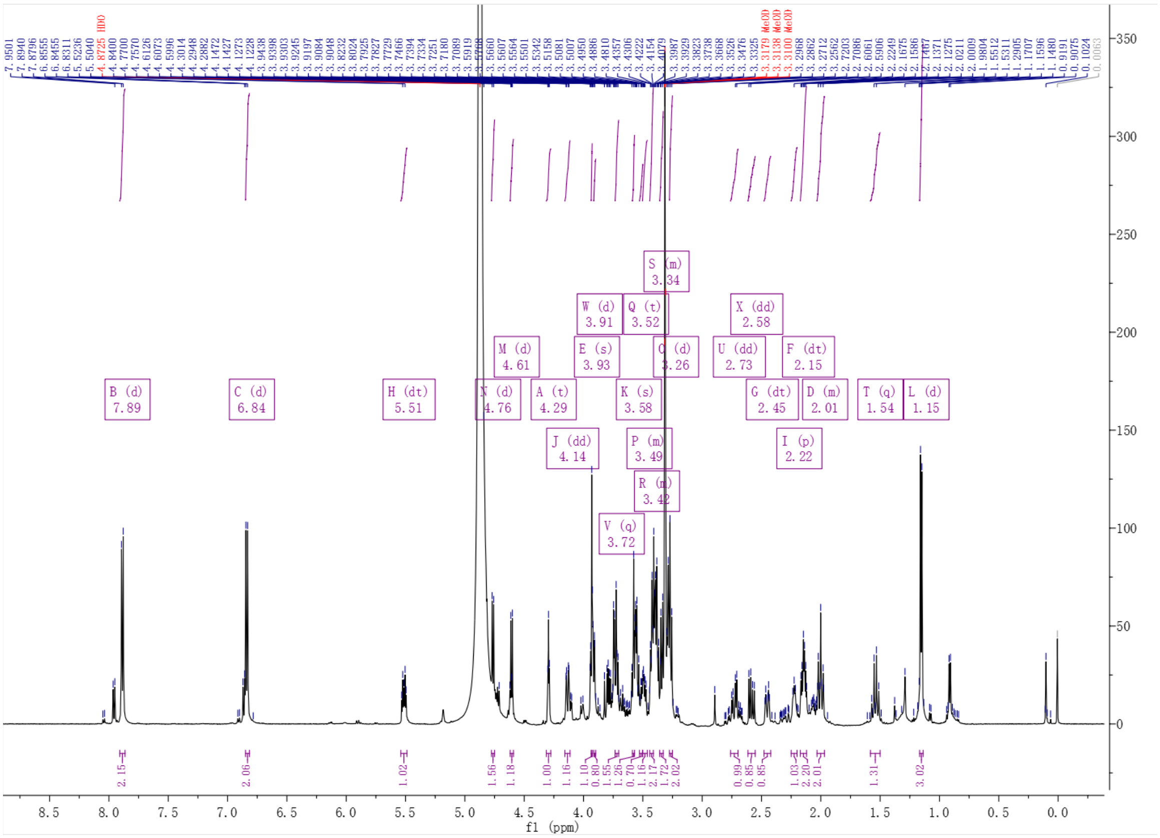

Figure S1. ^{13}C NMR spectrum of compound **1**

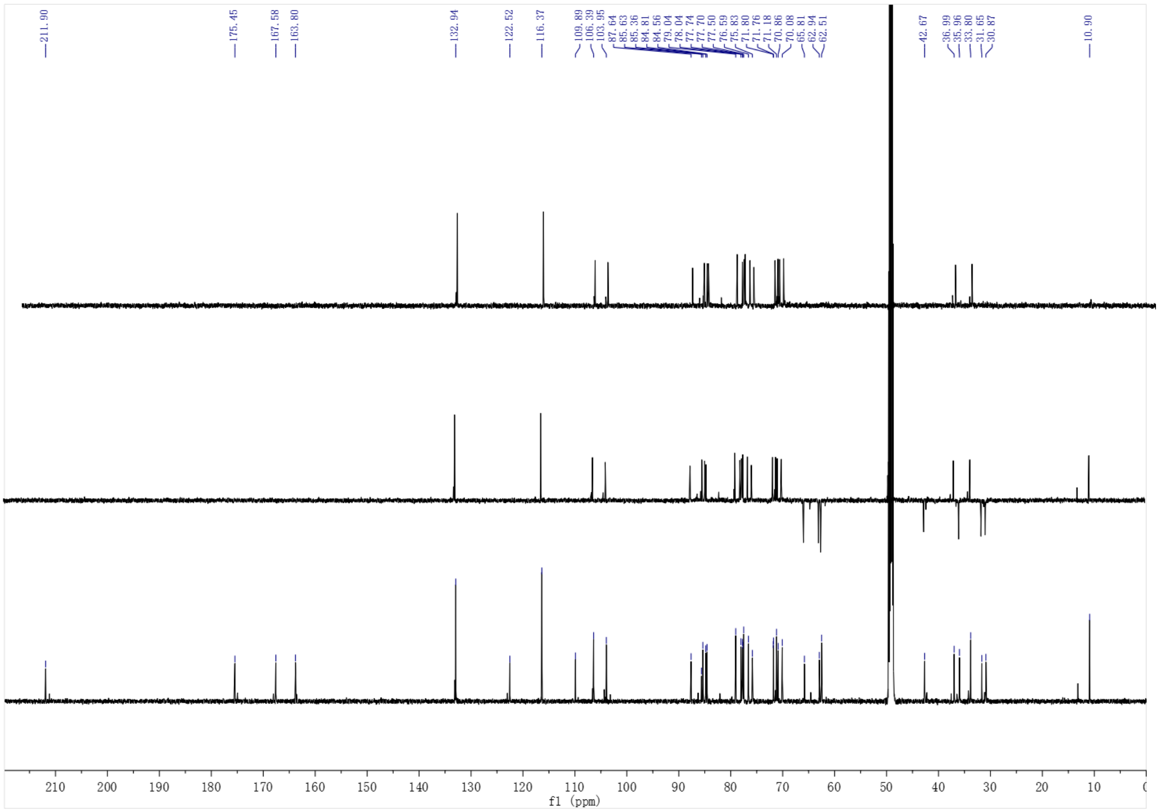

Figure S2. HSQC spectrum of compound **1**

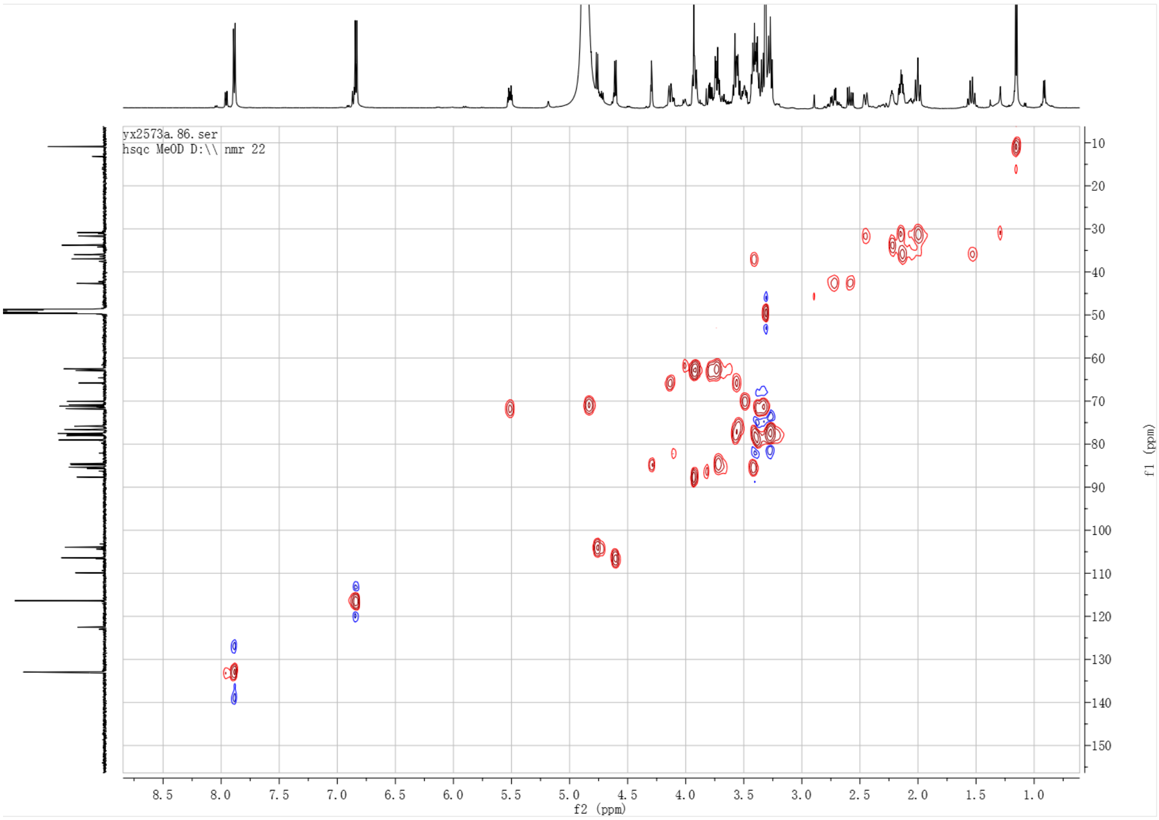

Figure S3. HMBC spectrum of compound **1**

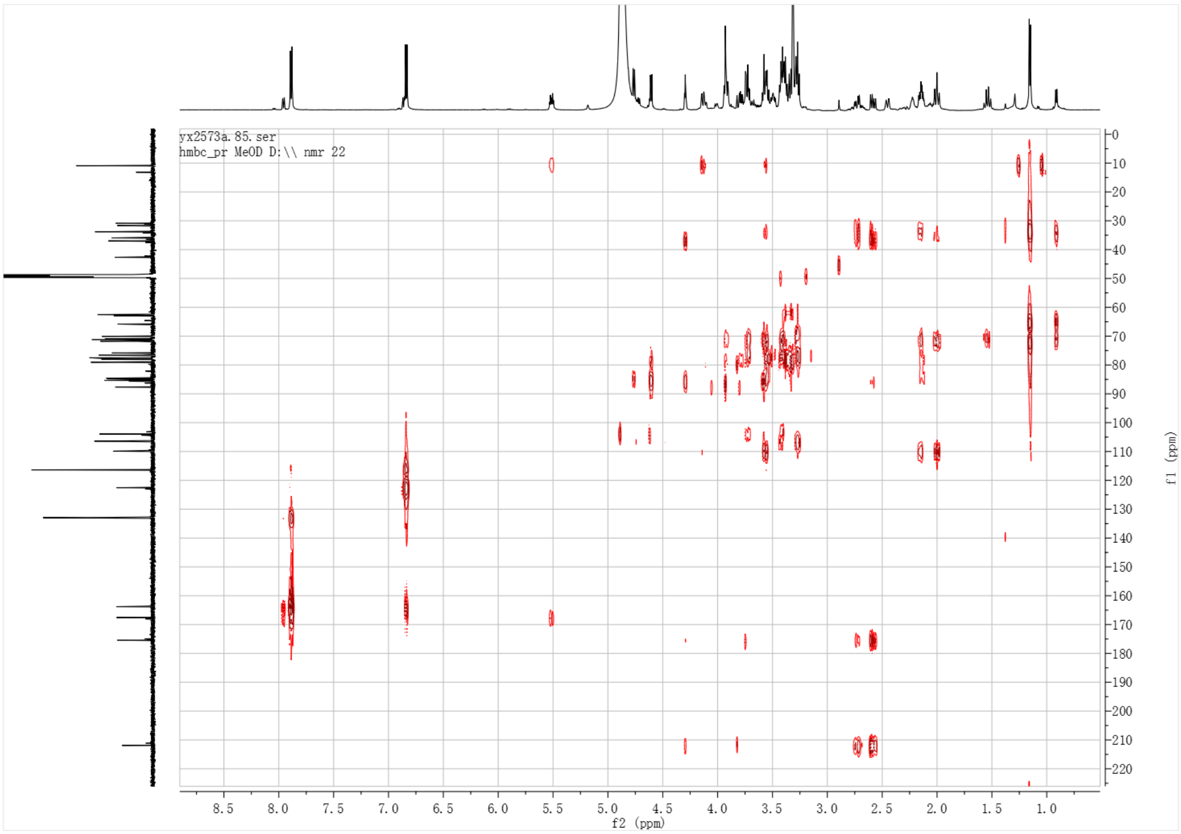

Figure S4. ^{1}H-^{1}H COSY spectrum of compound **1**

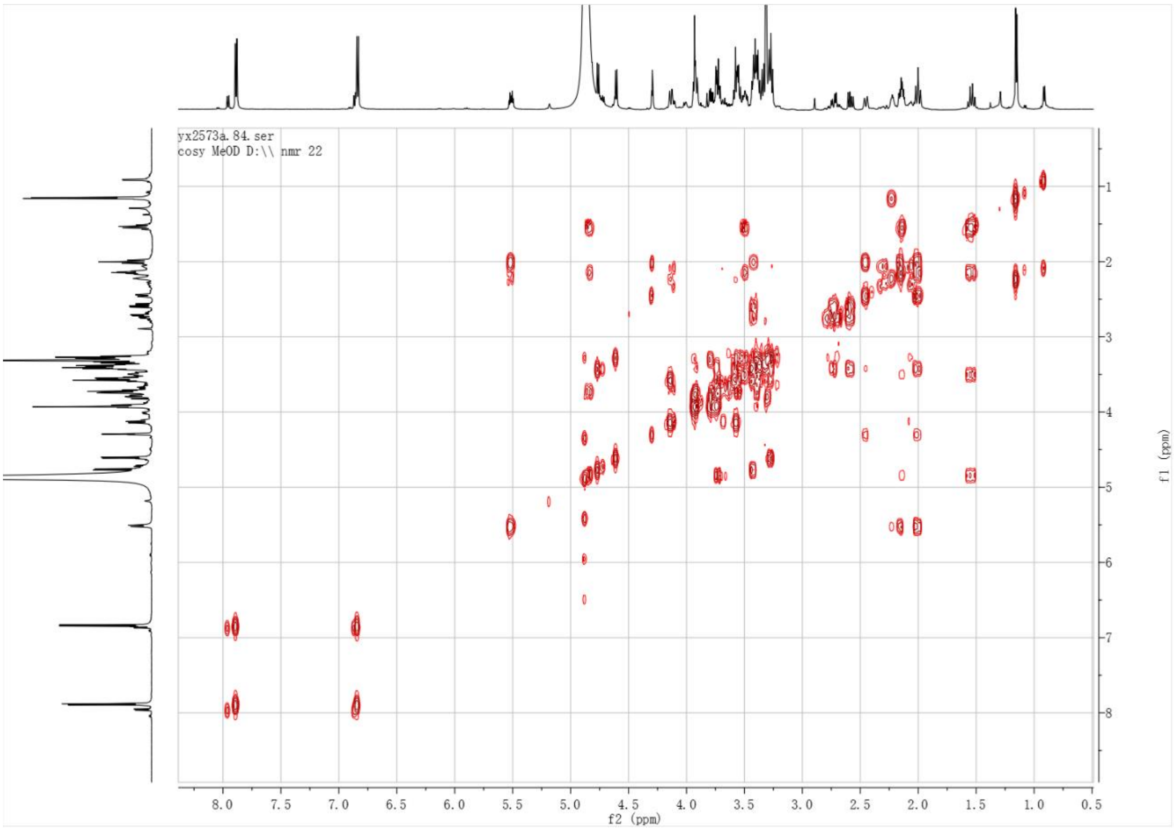

Figure S5. ROESY spectrum of compound **1**

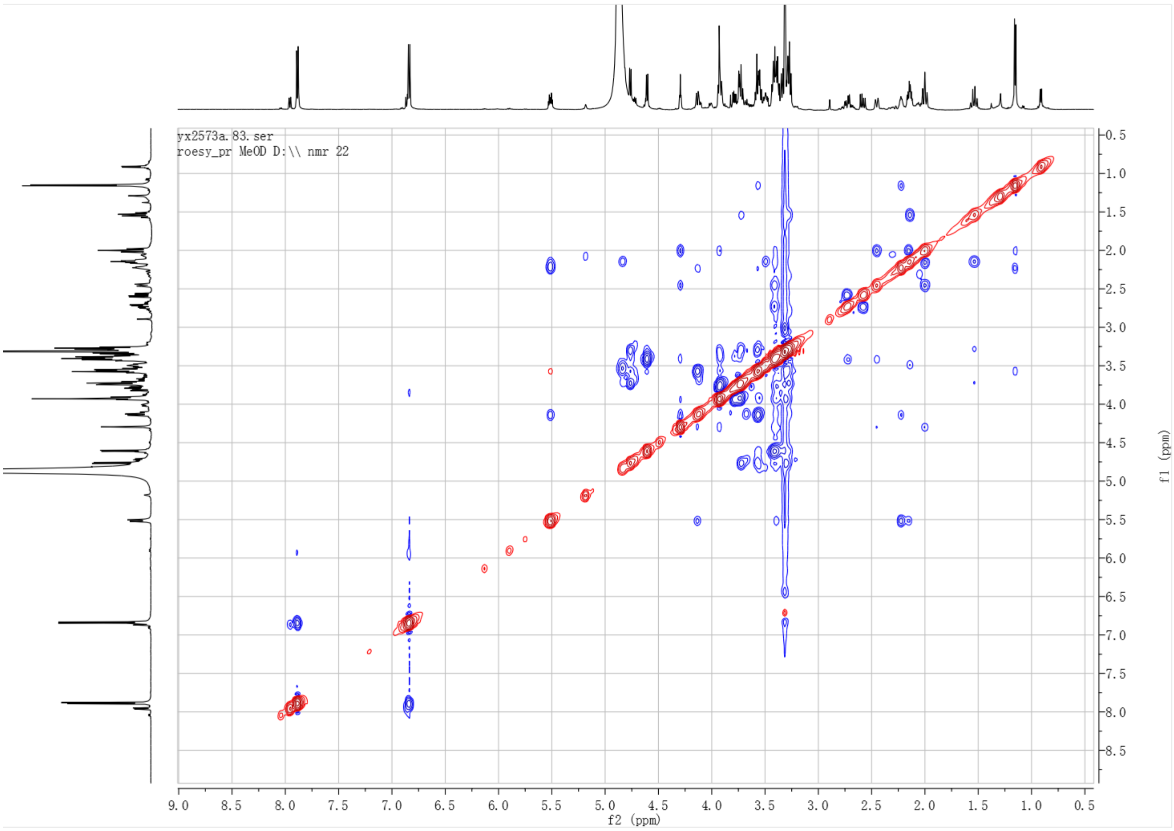

Figure S6. Negative ESIMS spectrum of compound **1**

Qualitative Analysis Report

**Data Filename**	yx-2-57-3.d	**Sample Name**	yx-2-57-3
**Sample Type**	Sample	**Position**	P1-B2
**Instrument Name**	Instrument 1	**User Name**	
**Acq Method**	s-.m	**Acquired Time**	8/25/2021 11:32:17 AM
**IRM Calibration Status**	Success	**DA Method**	Default.m
**Comment**			

Sample Group **Info.**

Acquisition SW Version 6200 series TOF/6500 series Q-TOF B.05.01 (B5125.2)

User Spectra

**Fragmentor Voltage**	**Collision Energy**	**Ionization Mode**
135	0	ESI

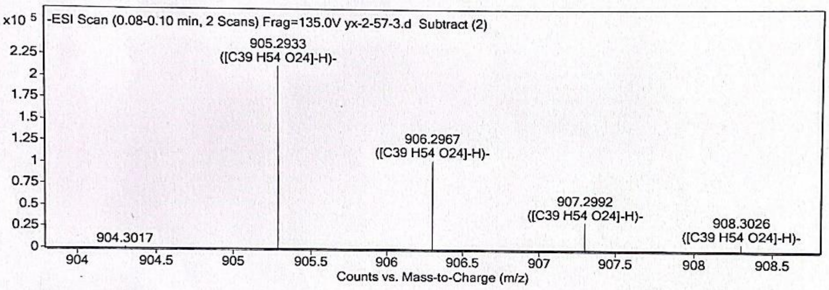

Peak List

m/z	z	Abund	Formula	Ion
154.9734	1	21787.93		
248.96	1	17059.3		
905.2933	1	213123.8	C39 H54 O24	(M-H)-
906.2967	1	104132.67	C39 H54 O24	(M-H)-
907.2992	1	31983.61	C39 H54 O24	(M-H)-
908.3026	1	7865.64	C39 H54 O24	(M-H)-
941.2691	1	39671.45		
942.2727	1	16956.48		
943.2697	1	17153.01		
973.2803	1	7750.23		

Formula Calculator Element Limits

Element	Min	Max
C	3	60
H	0	120
O	0	30

Formula Calculator Results

Formula	CalculatedMass	CalculatedMz	Mz	Diff. (mDa)	Diff. (ppm)	DBE
C39 H54 O24	906.3005	905.2932	905.2933	-0.10	-0.11	13.0000

--- End Of Report ---

Agilent Technologies Page 1 of 1 Printed at: 3:43 PM on: 8/27/2021

Figure S7. HRESIMS spectrum of compound **1**

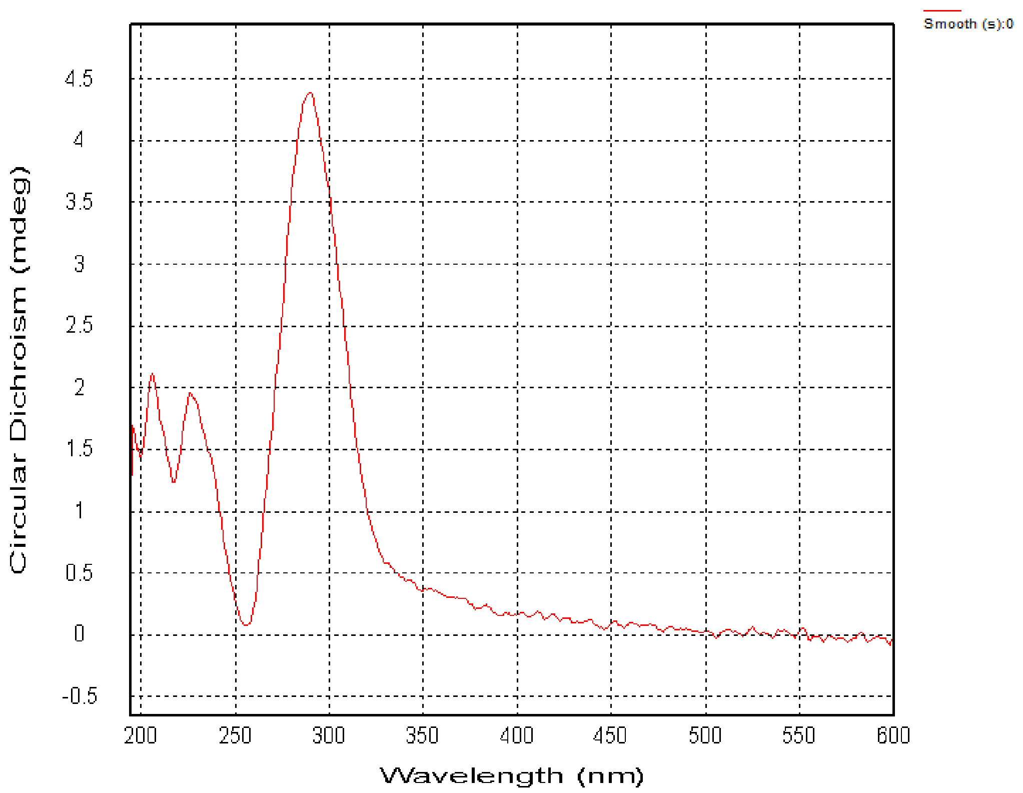

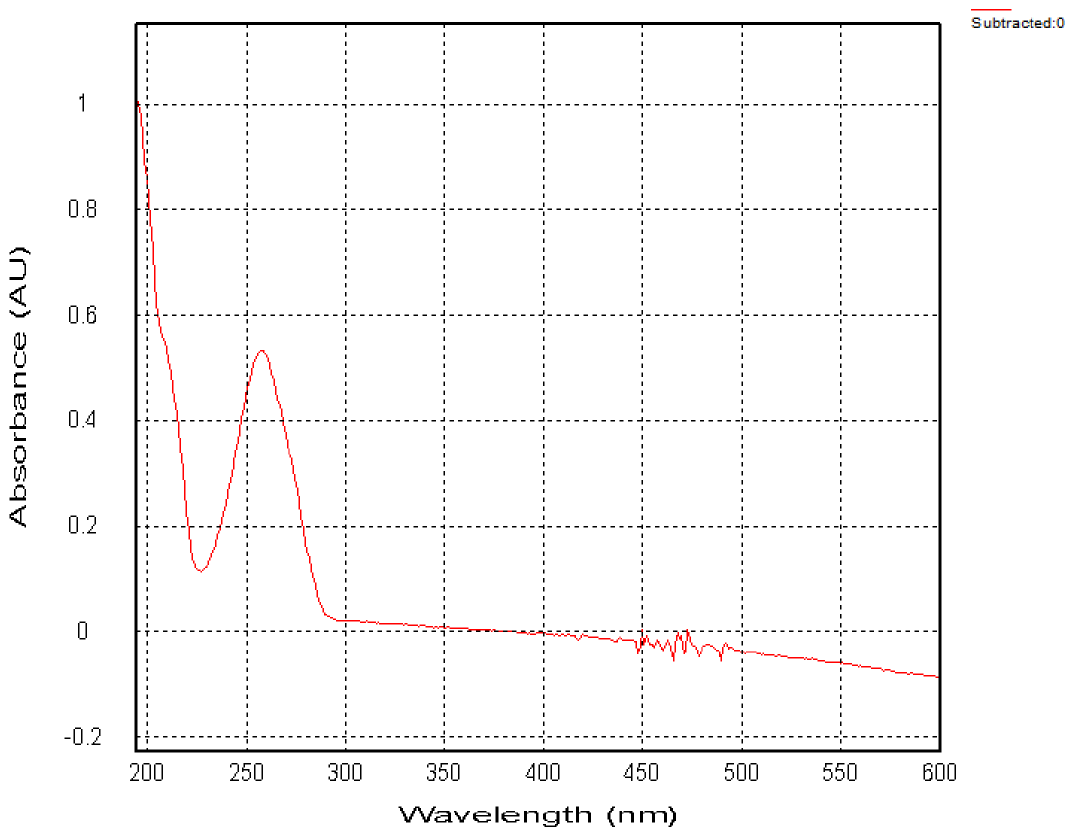

Figure S8. CD and UV spectra of compound **1**

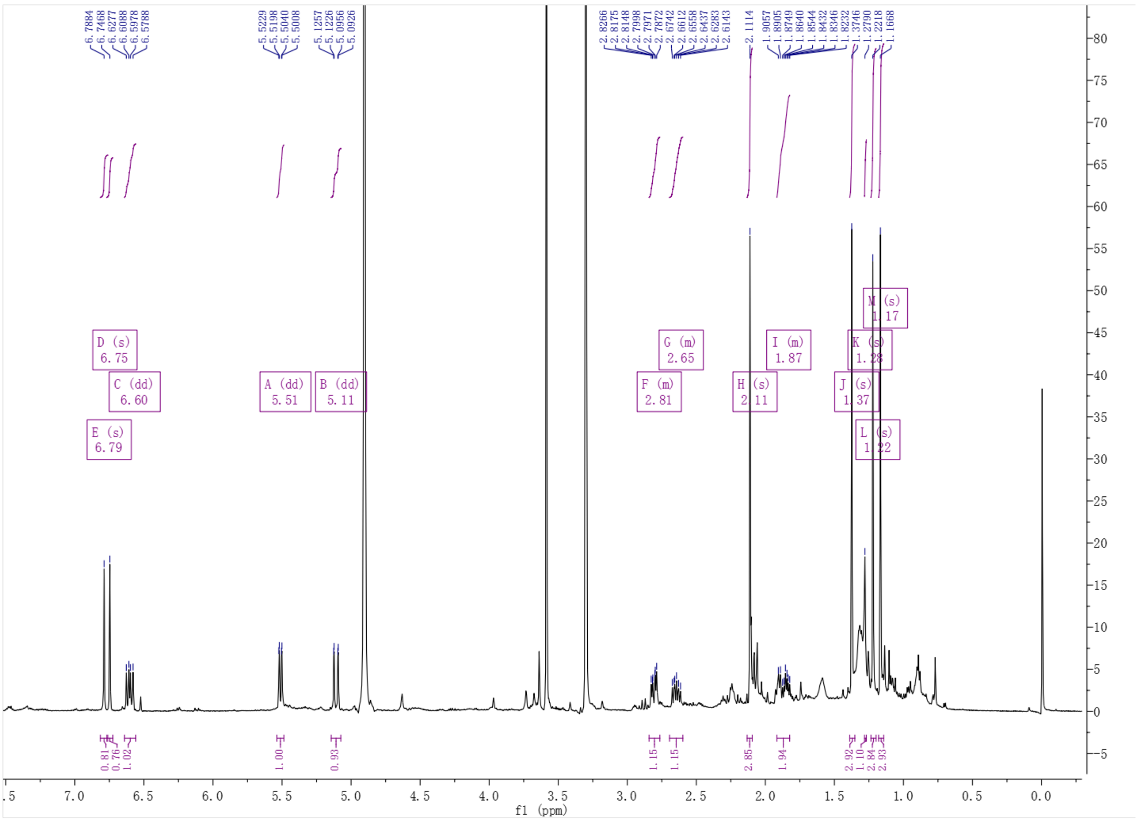

Figure S9. ^{1}H NMR spectrum of compound **2**

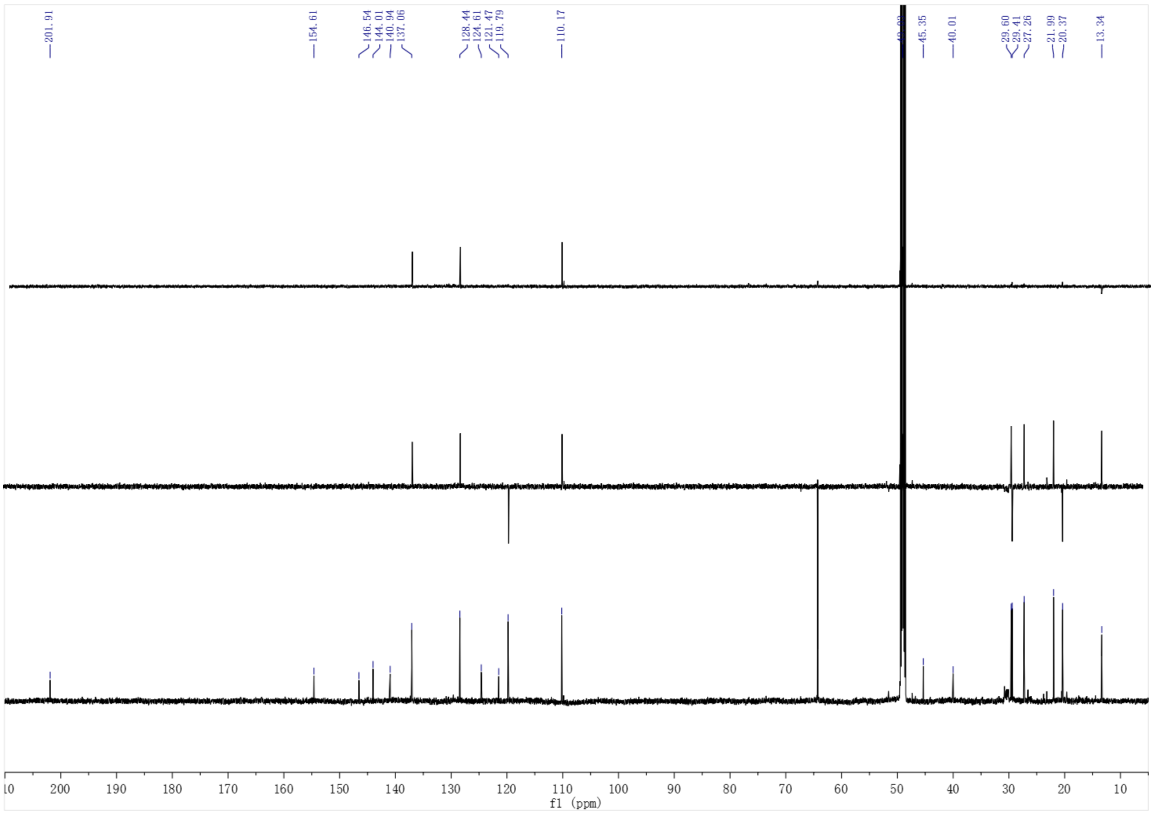

Figure S10. ^{13}C NMR spectrum of compound **2**

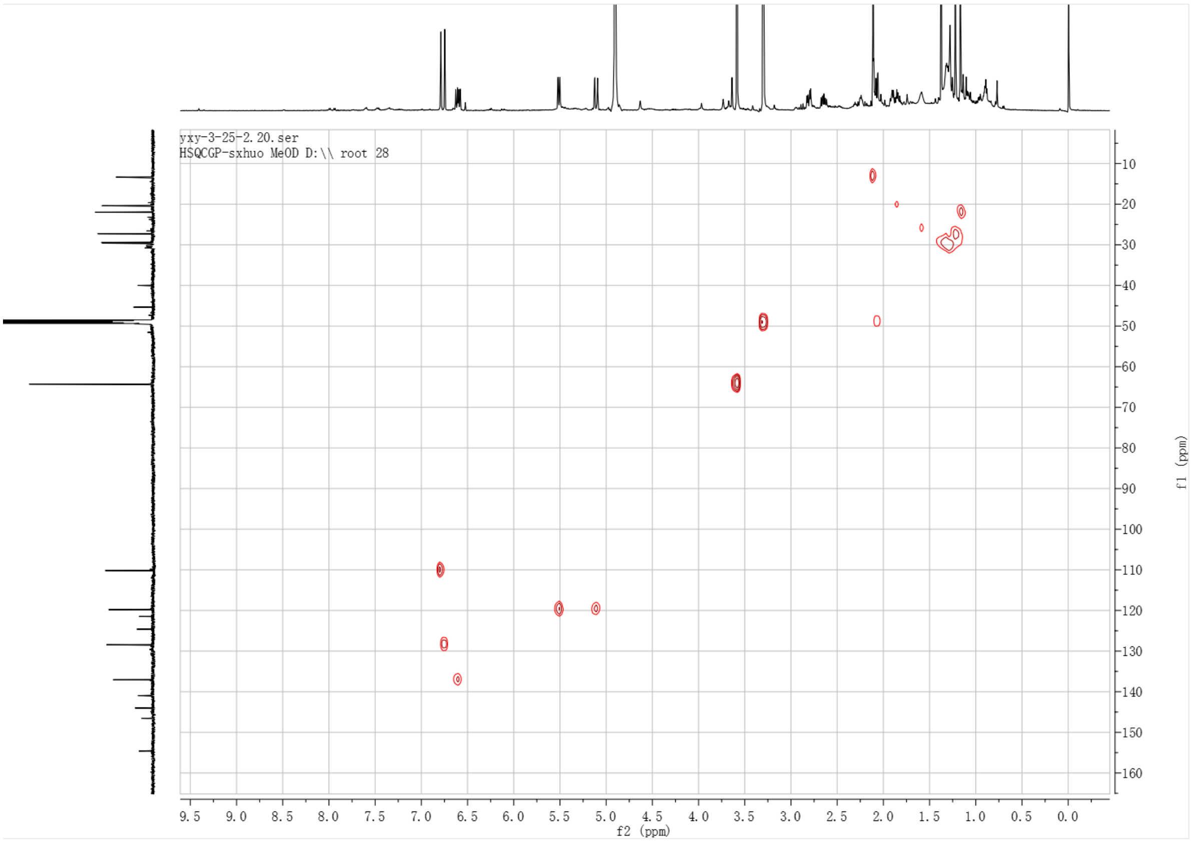

Figure S11. HSQC spectrum of compound **2**

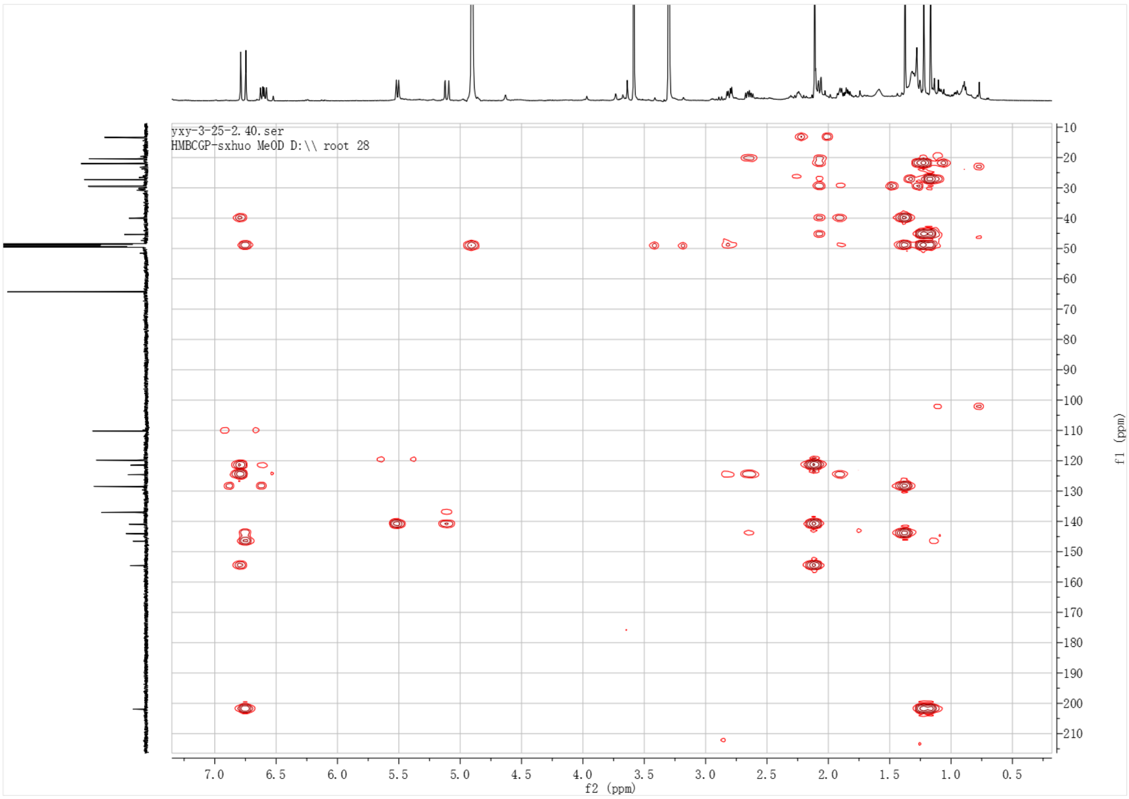

Figure S12. HMBC spectrum of compound **2**

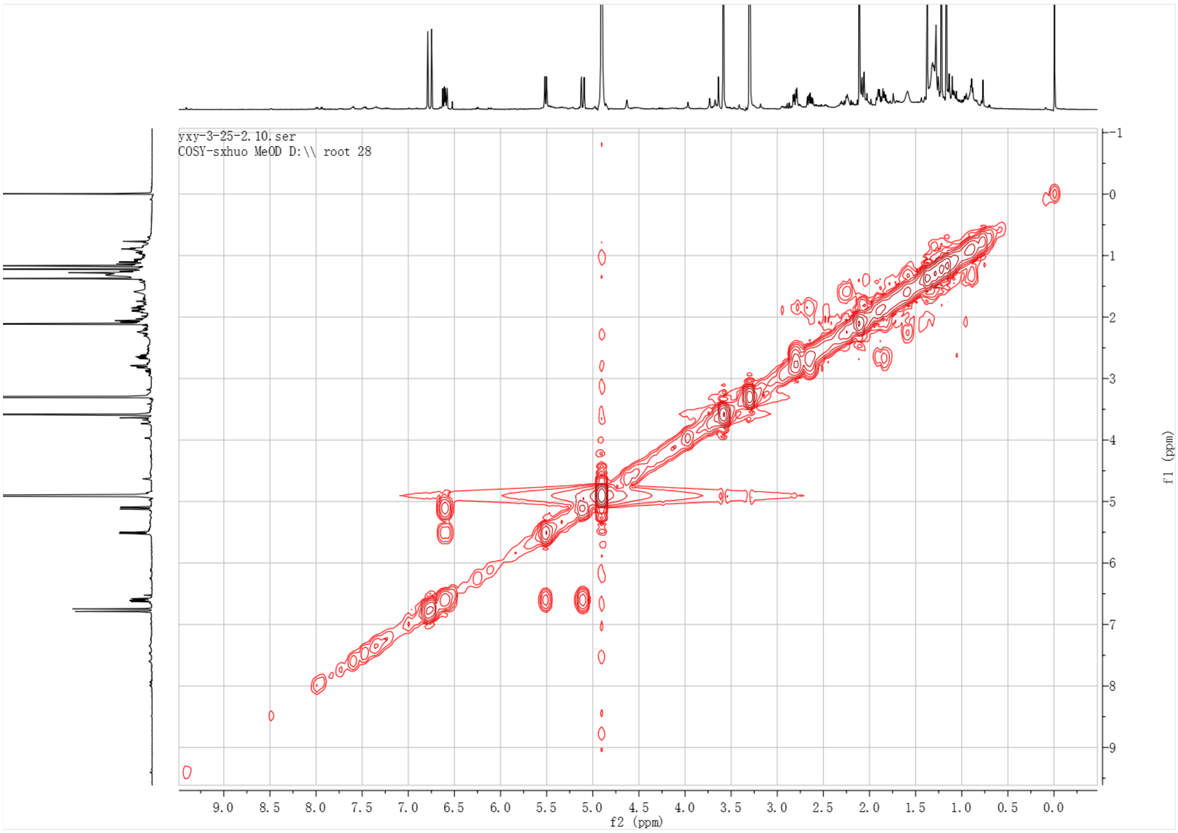

Figure S13. ^{1}H-^{1}H COSY spectrum of compound **2**

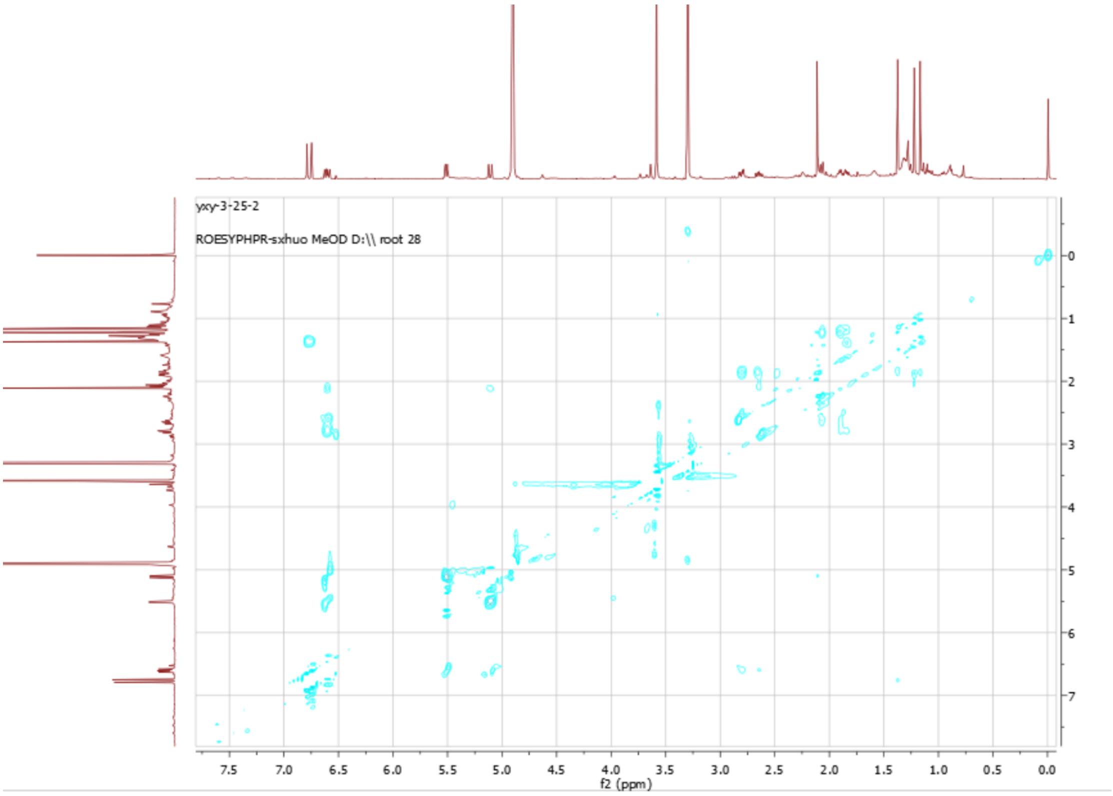

Figure S14. ROESY spectrum of compound **2**

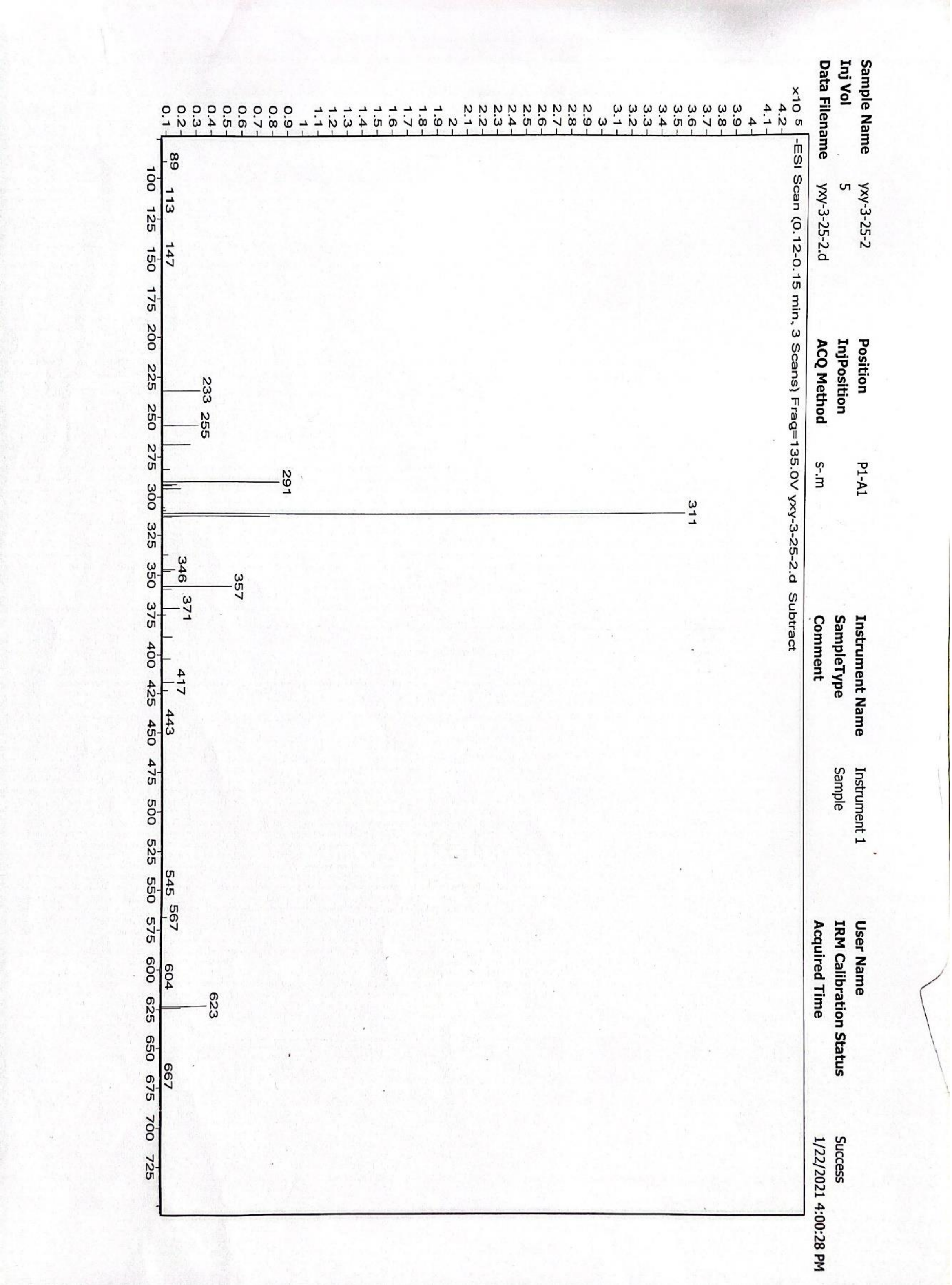

Figure S15. Negative ESIMS spectrum of compound **2**

Qualitative Analysis Report

**Data Filename**	yxy-3-25-2.d	**Sample Name**	yxy-3-25-2
**Sample Type**	Sample	**Position**	P1-A1
**Instrument Name**	Instrument 1	**User Name**	
**Acq Method**	s-.m	**Acquired Time**	1/22/2021 4:06:11 PM
**IRM Calibration Status**	Success	**DA Method**	Default.m
**Comment**			

Sample Group **Info.**

Acquisition SW Version 6200 series TOF/6500 series Q-TOF B.05.01 (B5125.2)

User Spectra

Fragmentor Voltage	Collision Energy	Ionization Mode
135	0	ESI

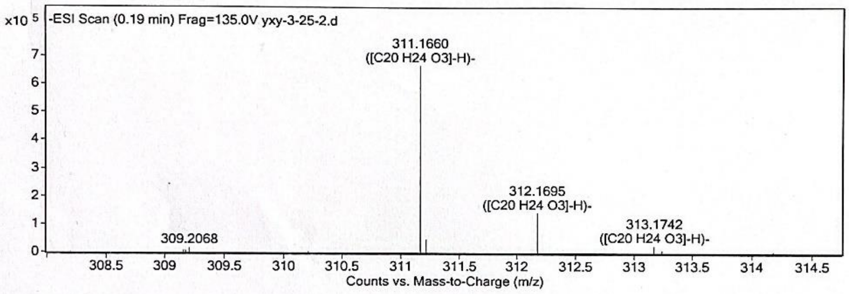

Peak List

m/z	z	Abund	Formula	Ion
89.0244		55099.21		
233.1546	1	61750.66		
255.2332	1	60509.23		
267.1968	1	55866.35		
291.1969	1	171677.63		
311.166	1	668953.44	C20 H24 O3	(M-H)-
312.1695	1	141789.66	C20 H24 O3	(M-H)-
357.1713	1	88015.73		
623.3383	1	94198.52		
624.3422	1	56831.93		

Formula Calculator Element Limits

Element	Min	Max
C	3	60
H	0	120
O	0	30
Cl	0	3

Formula Calculator Results

Formula	CalculatedMass	CalculatedMz	Mz	Diff. (mDa)	Diff. (ppm)	DBE
C20 H24 O3	312.1725	311.1653	311.1660	-0.70	-2.25	9.0000

--- End Of Report ---

Agilent Technologies Page 1 of 1 Printed at: 3:21 PM on: 1/26/2021

Figure S16. HRESIMS spectrum of compound **2**

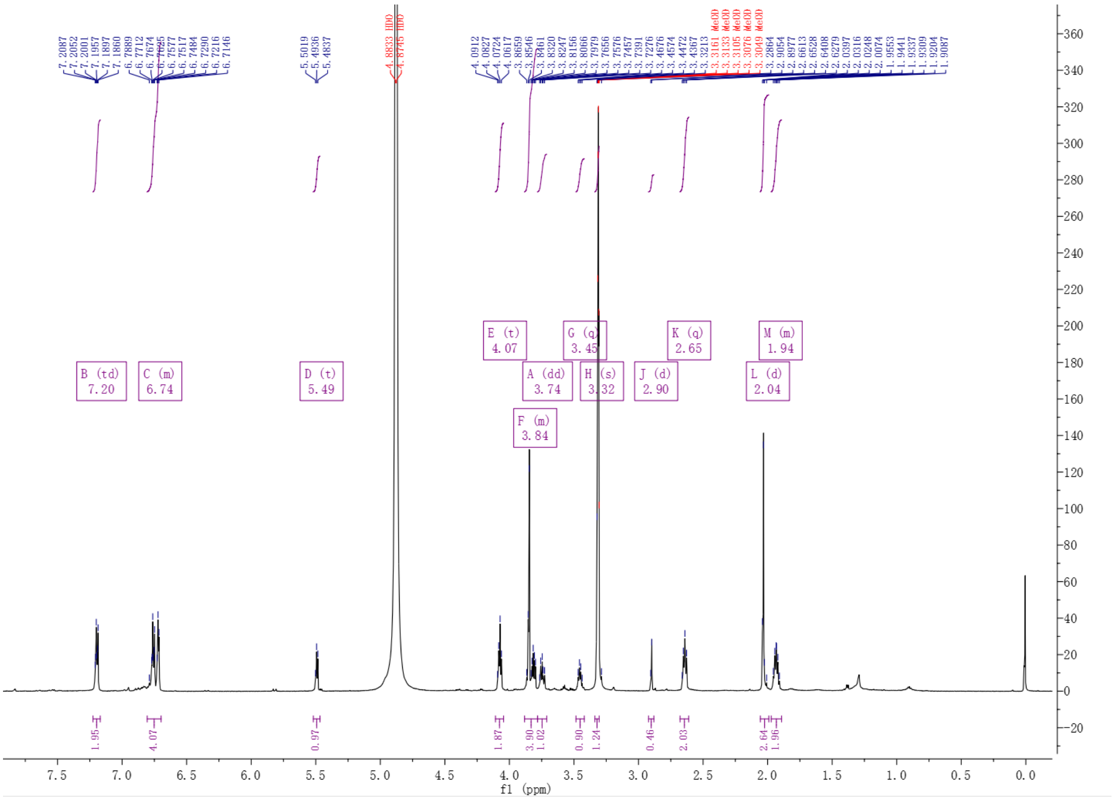

Figure S17. ^{1}H NMR spectrum of compound **3**

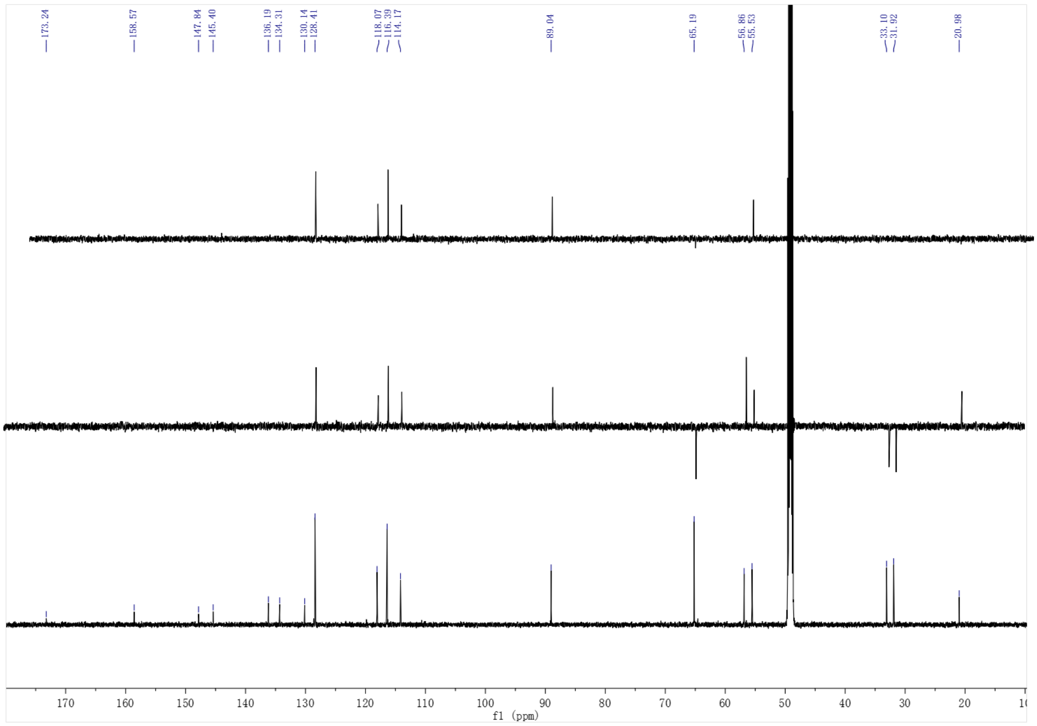

Figure S18. ^{13}C NMR spectrum of compound **3**

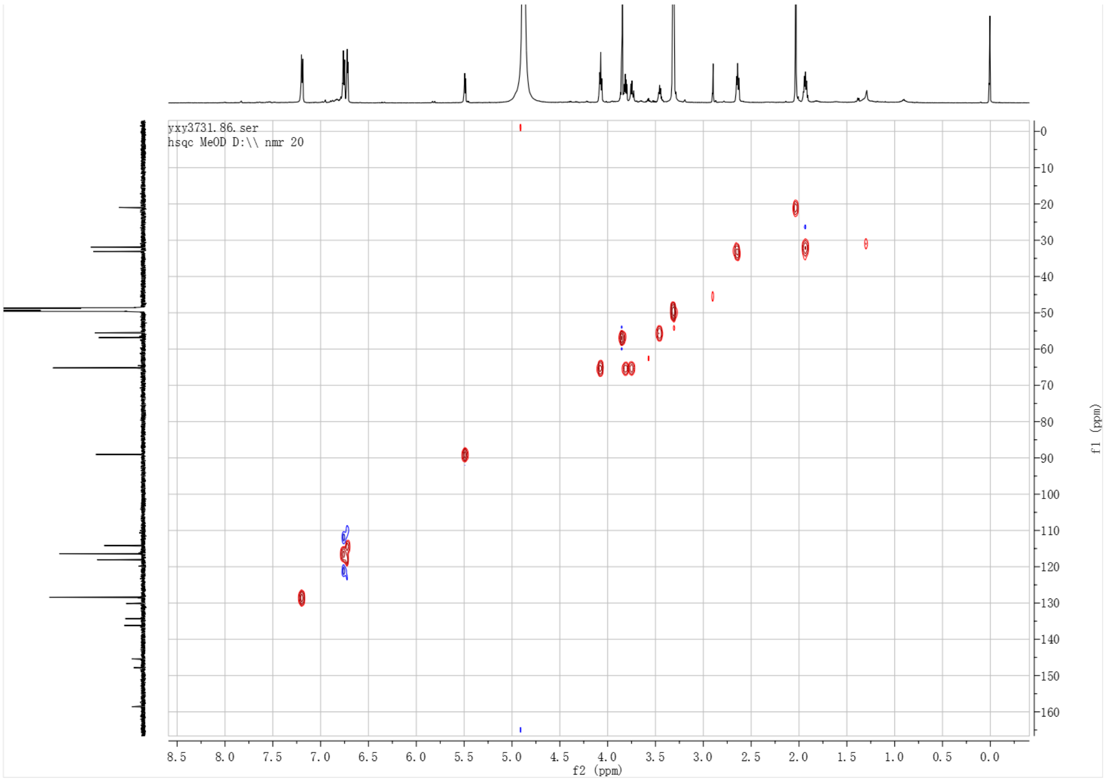

Figure S19. HSQC spectrum of compound **3**

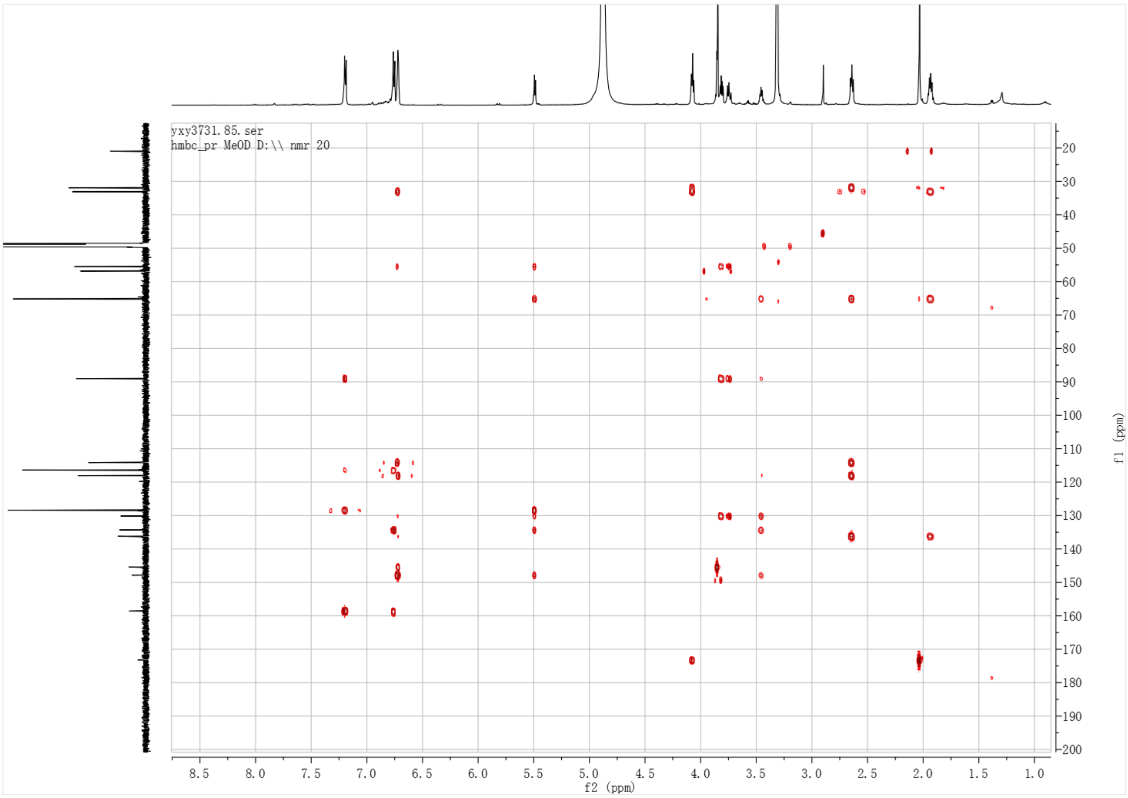

Figure S20. HMBC spectrum of compound **3**

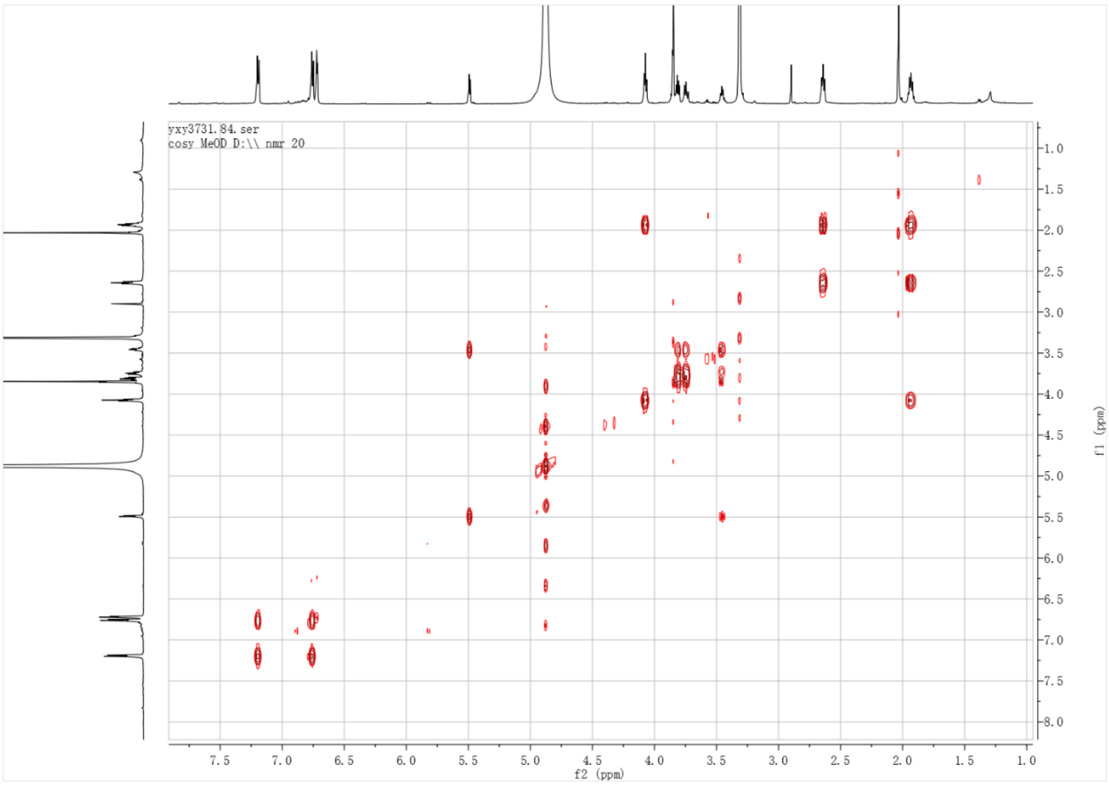

Figure S21. ^{1}H-^{1}H COSY spectrum of compound **3**

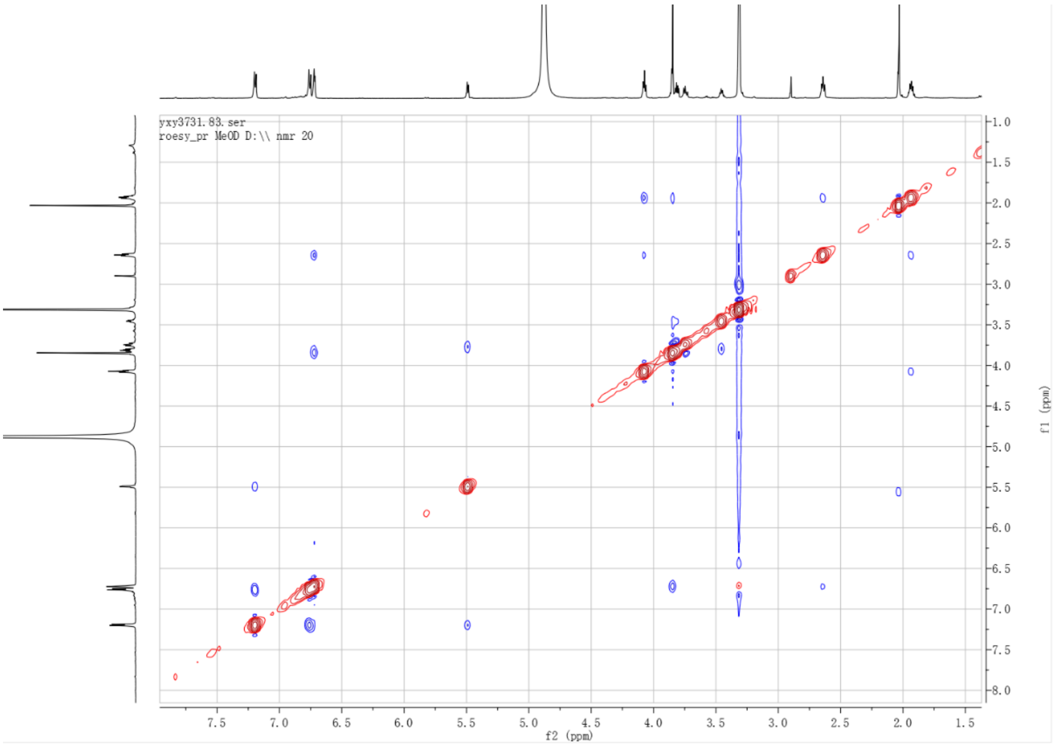

Figure S22. ROESY spectrum of compound **3**

Qualitative Analysis Report

**Data Filename**	yxy-3-73-1.d	**Sample Name**	yxy-3-73-1
**Sample Type**	Sample	**Position**	P1-A3
**Instrument Name**	Instrument 1	**User Name**	
**Acq Method**	s-.m	**Acquired Time**	8/26/2021 4:17:10 PM
**IRM Calibration Status**	Success	**DA Method**	Default.m
**Comment**			

Sample Group

Info.

Acquisition SW Version 6200 series TOF/6500 series Q-TOF B.05.01 (B5125.2)

User Spectra

Fragmentor Voltage	Collision Energy	Ionization Mode
135	0	ESI

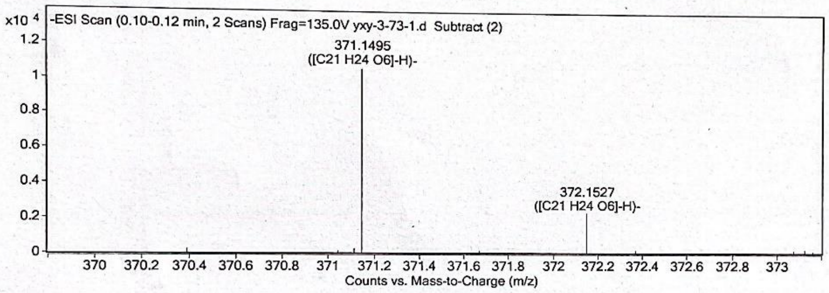

Peak List

m/z	z	Abund	Formula	Ion
293.0815	1	16712.16		
299.1285	1	3197.31		
341.139	1	83773.43		
342.1423	1	18094.04		
353.1392	1	58272.19		
354.1427	1	12563.1		
371.1495	1	10647.33	C21 H24 O6	(M-H)-
375.2749	1	9360.27		
403.306	1	12966.62		
417.1539		4513.54		

Formula Calculator Element Limits

Element	Min	Max
C	3	60
H	0	120
O	0	30
N	0	10

Formula Calculator Results

Formula	CalculatedMass	CalculatedMz	Mz	Diff. (mDa)	Diff. (ppm)	DBE
C21 H24 O6	372.1573	371.1500	371.1495	0.50	1.35	10.0000

--- End Of Report ---

Agilent Technologies Page 1 of 1 Printed at: 11:20 AM on: 8/27/2021

Figure S23. HRESIMS spectrum of compound **3**

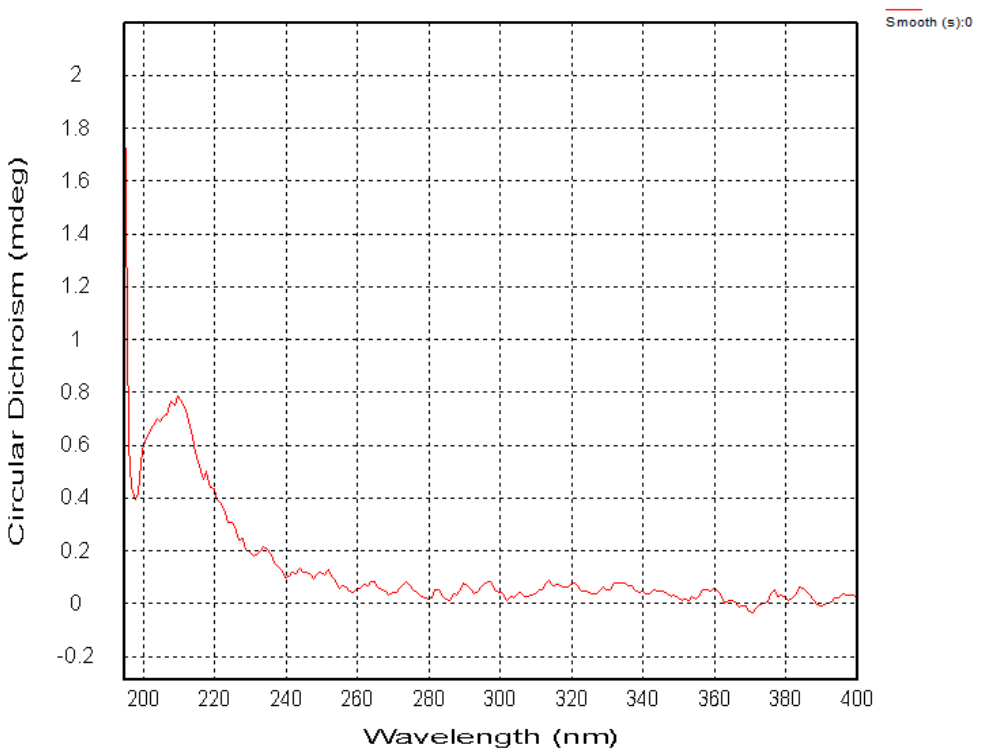

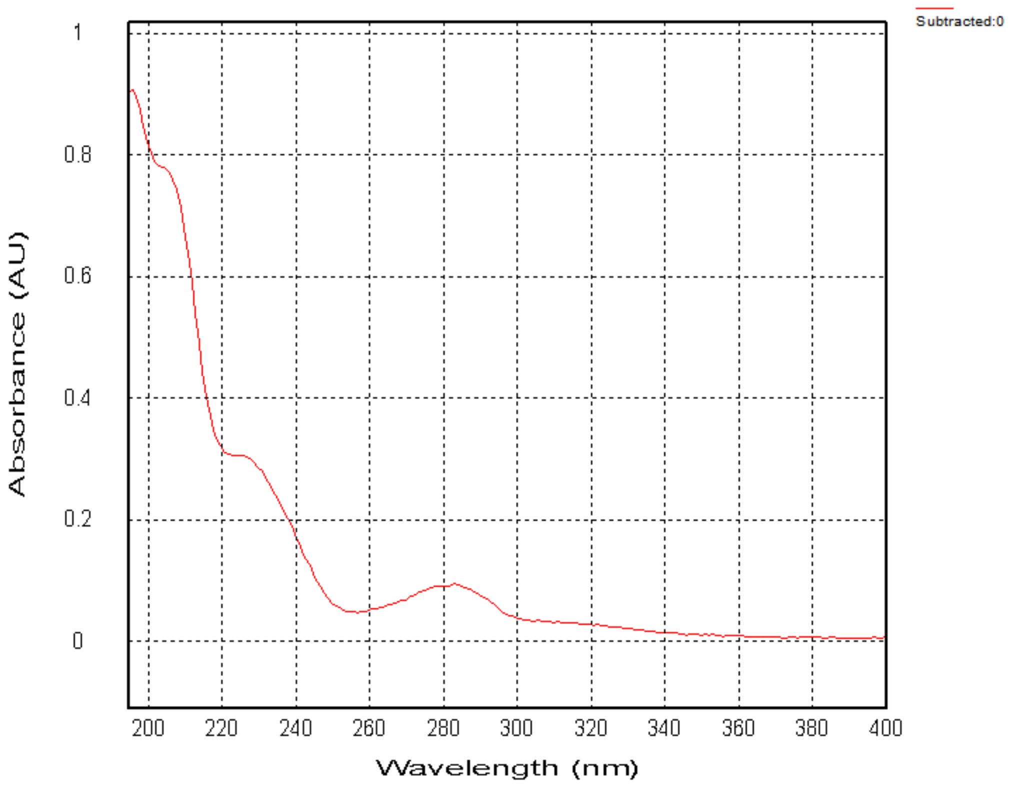

Figure S24. CD and UV spectra of compound **3**

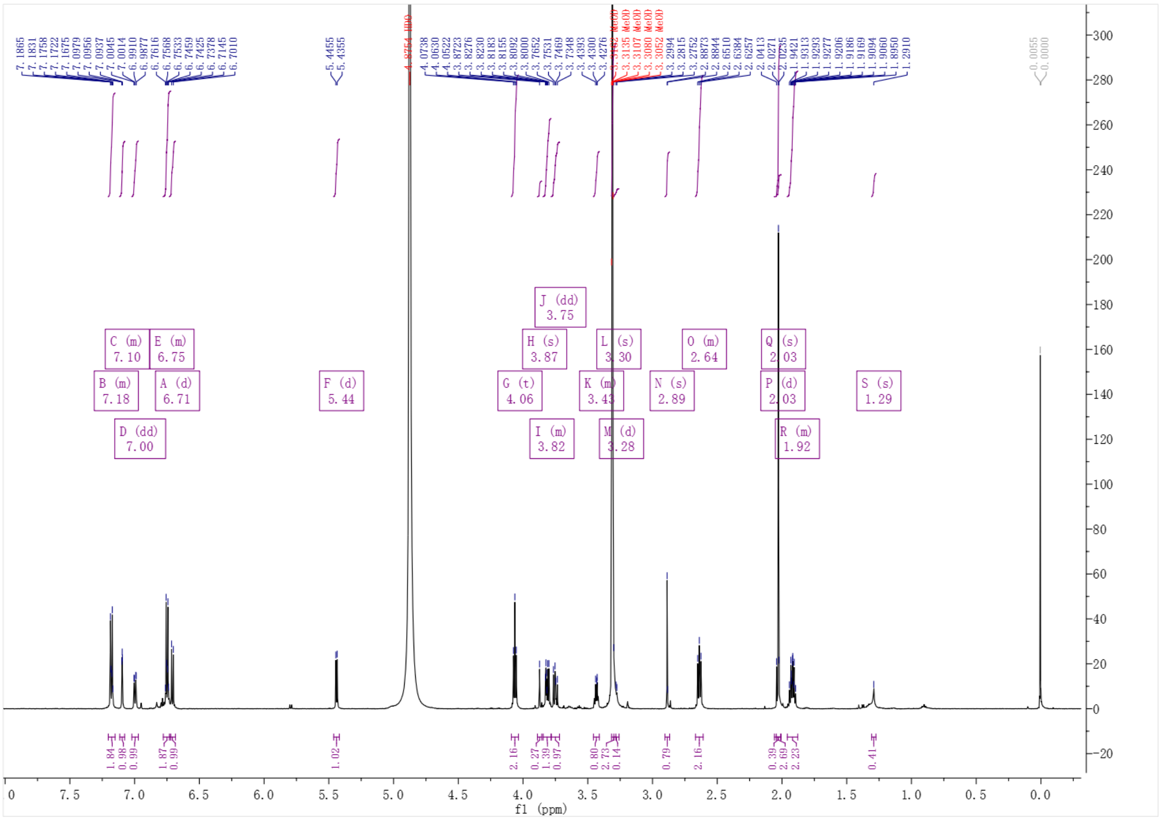

Figure S25. ^{1}H NMR spectrum of compound **4**

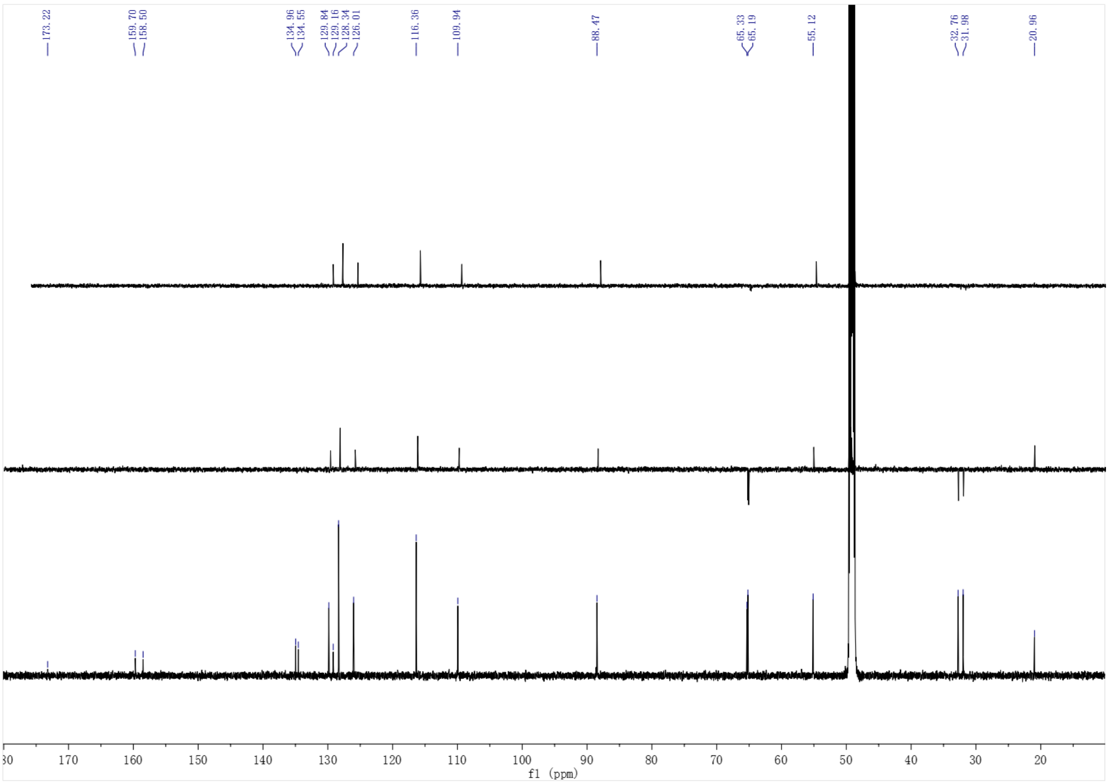

Figure S26. ^{13}C NMR spectrum of compound **4**

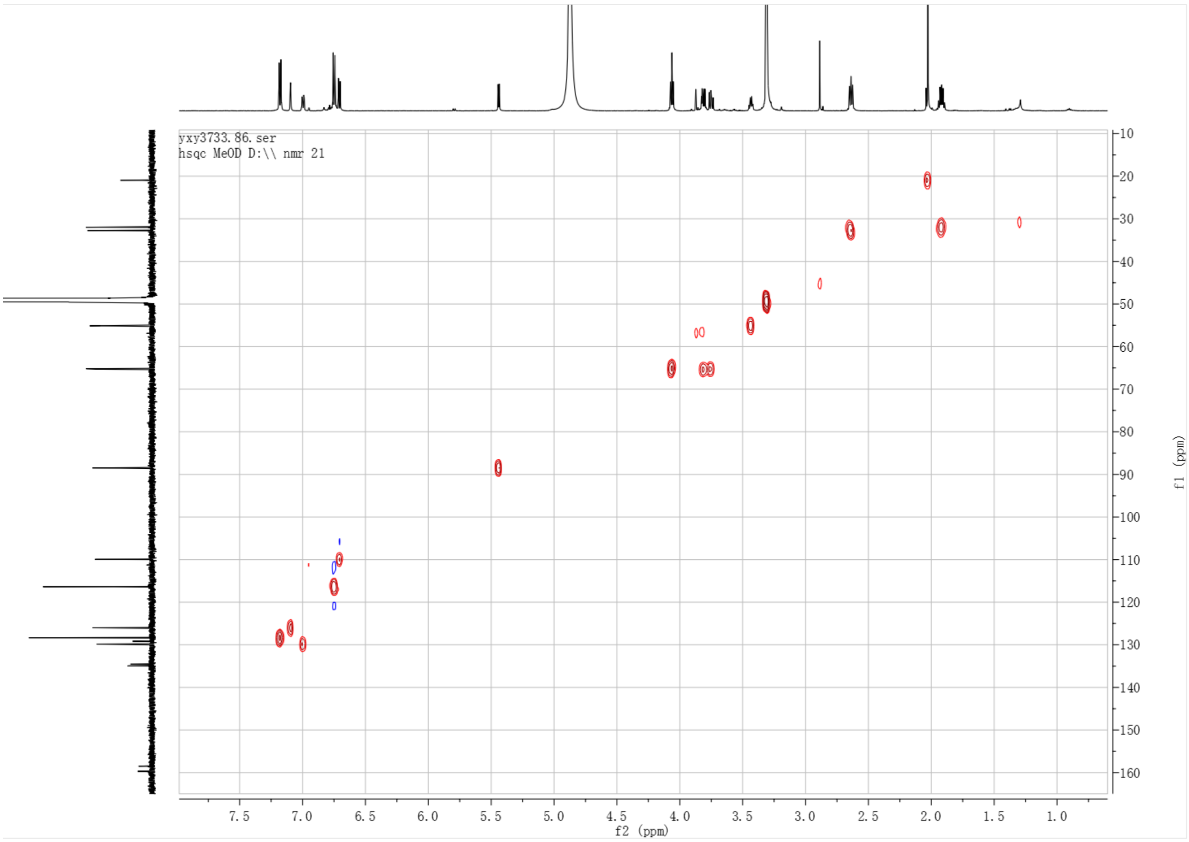

Figure S27. HSQC spectrum of compound **4**

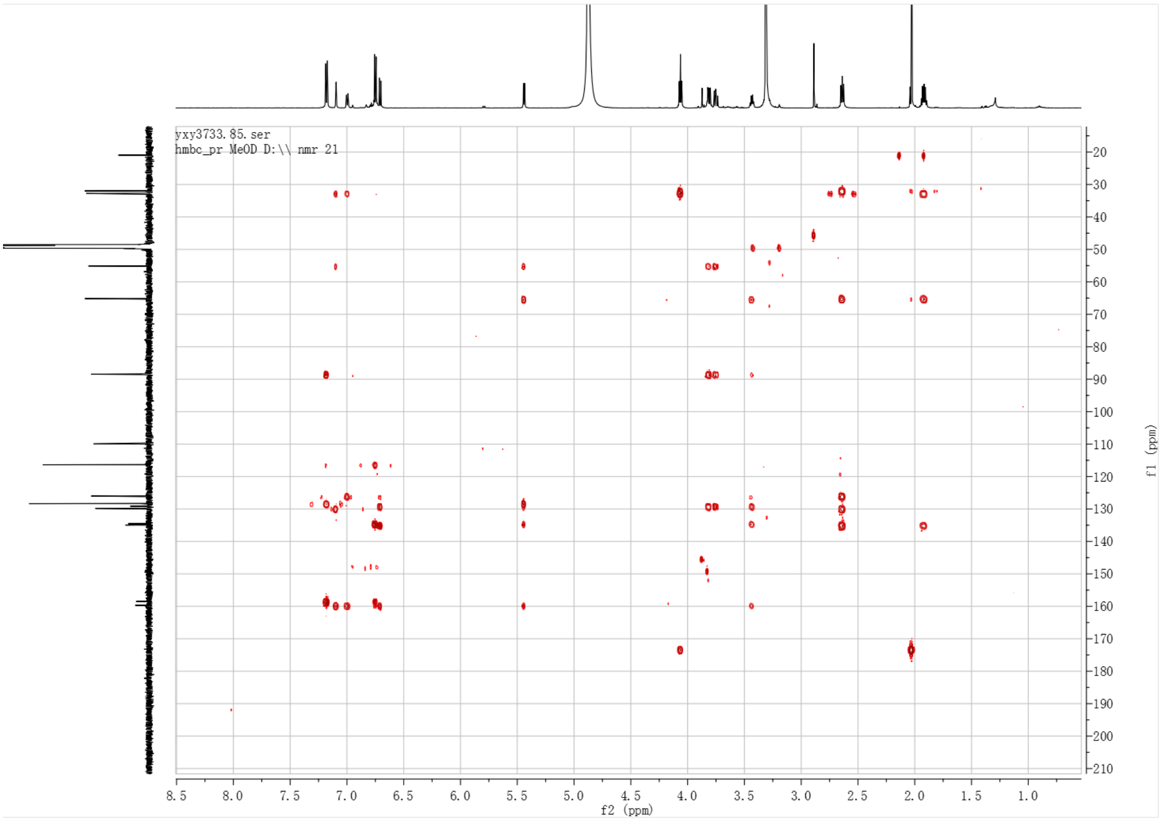

Figure S28. HMBC spectrum of compound **4**

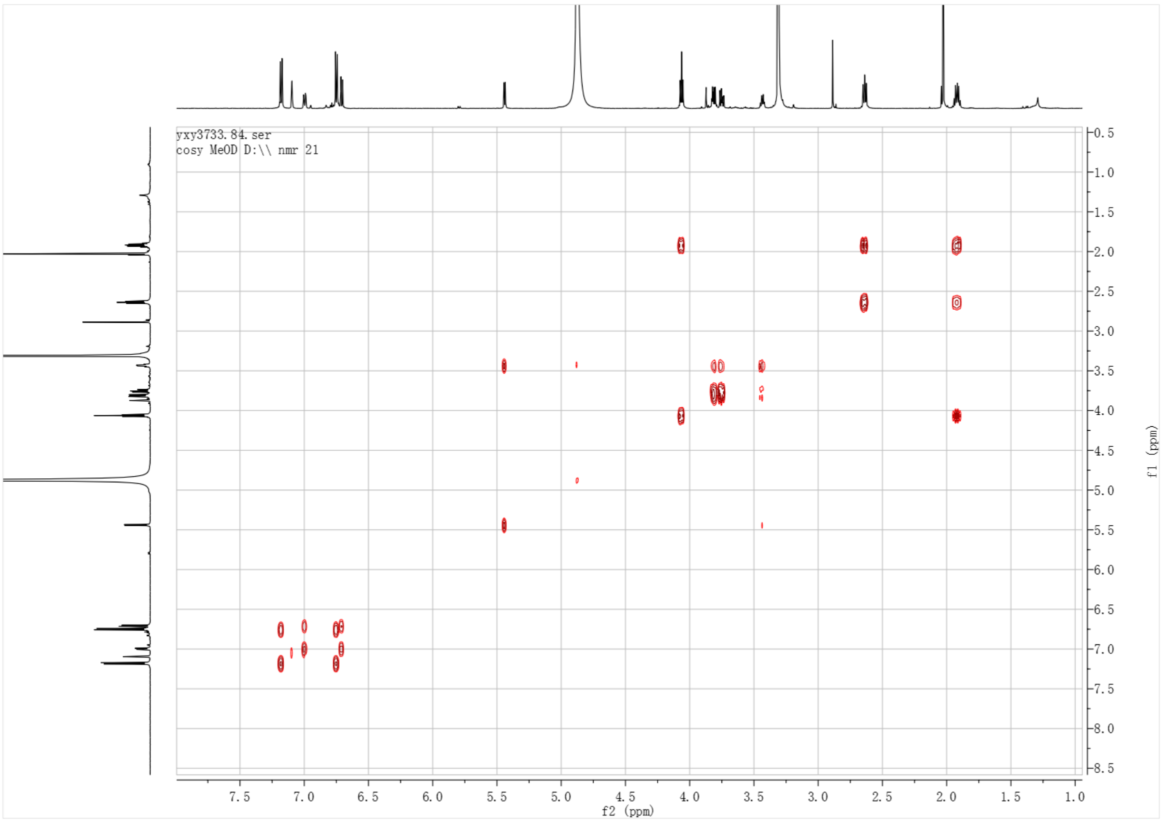

Figure S29. ^{1}H-^{1}H COSY spectrum of compound **4**

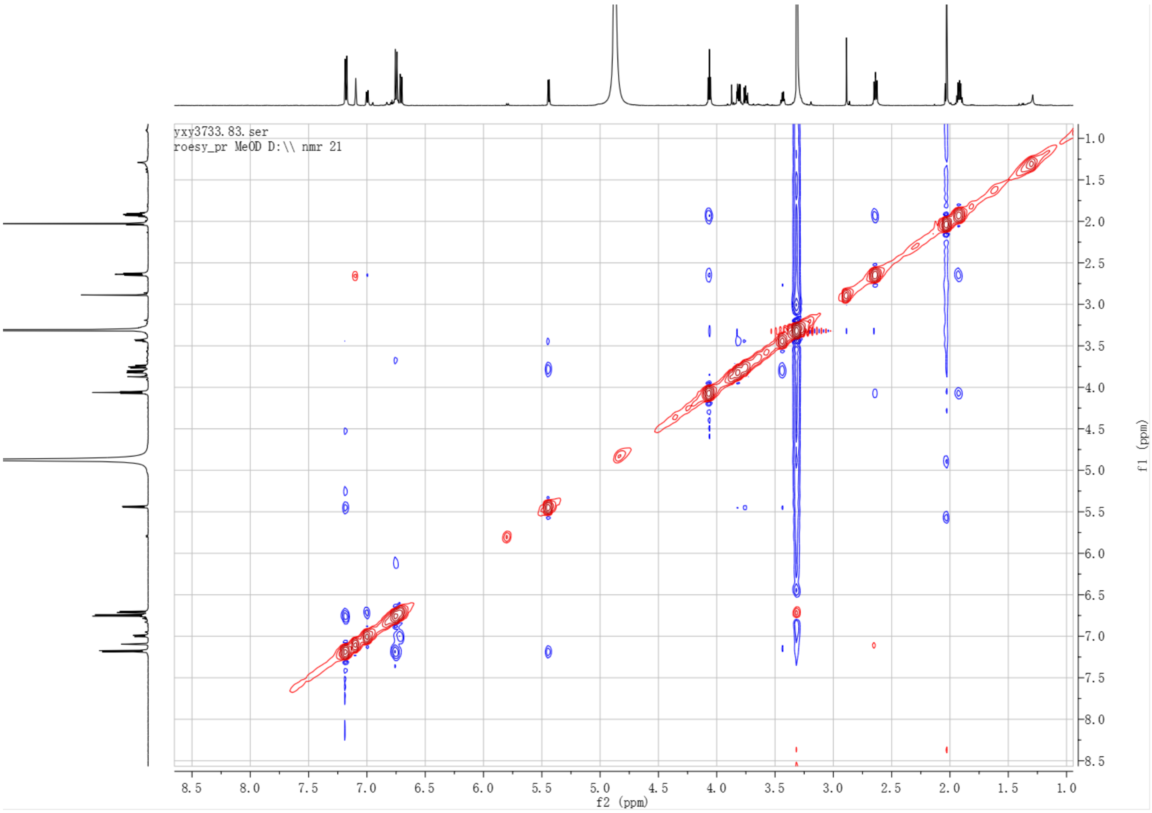

Figure S30. ROESY spectrum of compound **4**

Qualitative Analysis Report

**Data Filename**	yxy-3-73-3.d	**Sample Name**	yxy-3-73-3
**Sample Type**	Sample	**Position**	P1-A5
**Instrument Name**	Instrument 1	**User Name**	
**Acq Method**	s-.m	**Acquired Time**	8/26/2021 4:19:33 PM
**IRM Calibration Status**	Success	**DA Method**	Default.m
**Comment**			

Sample Group **Info.**

Acquisition SW Version 6200 series TOF/6500 series Q-TOF B.05.01 (B5125.2)

User Spectra

**Fragmentor Voltage**	**Collision Energy**	**Ionization Mode**
135	0	ESI

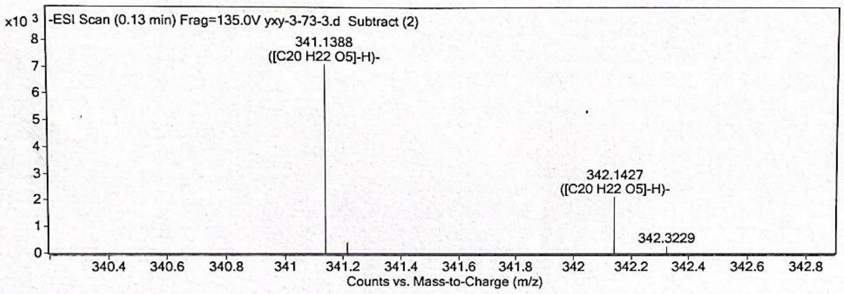

Peak List

m/z	z	Abund	Formula	Ion
311.1287	1	118646.88		
312.1321	1	23512.13		
323.1287	1	33275.08		
324.1317	1	8659.97		
341.1388	1	7094.22	C20 H22 O5	(M-H)-
375.2746	1	20926.98		
387.1434	1	4490.35		
393.2772	1	4622.18		
403.3063	1	28544.2		
404.309	1	6129.58		

Formula Calculator Element Limits

Element	Min	Max
C	3	60
H	0	120
O	0	30
N	0	10

Formula Calculator Results

Formula	CalculatedMass	CalculatedMz	Mz	Diff. (mDa)	Diff. (ppm)	DBE
C20 H22 O5	342.1467	341.1394	341.1388	0.60	1.76	10.0000

--- End Of Report ---

Agilent Technologies Page 1 of 1 Printed at: 11:21 AM on: 8/27/2021

Figure S31. HRESIMS spectrum of compound **4**

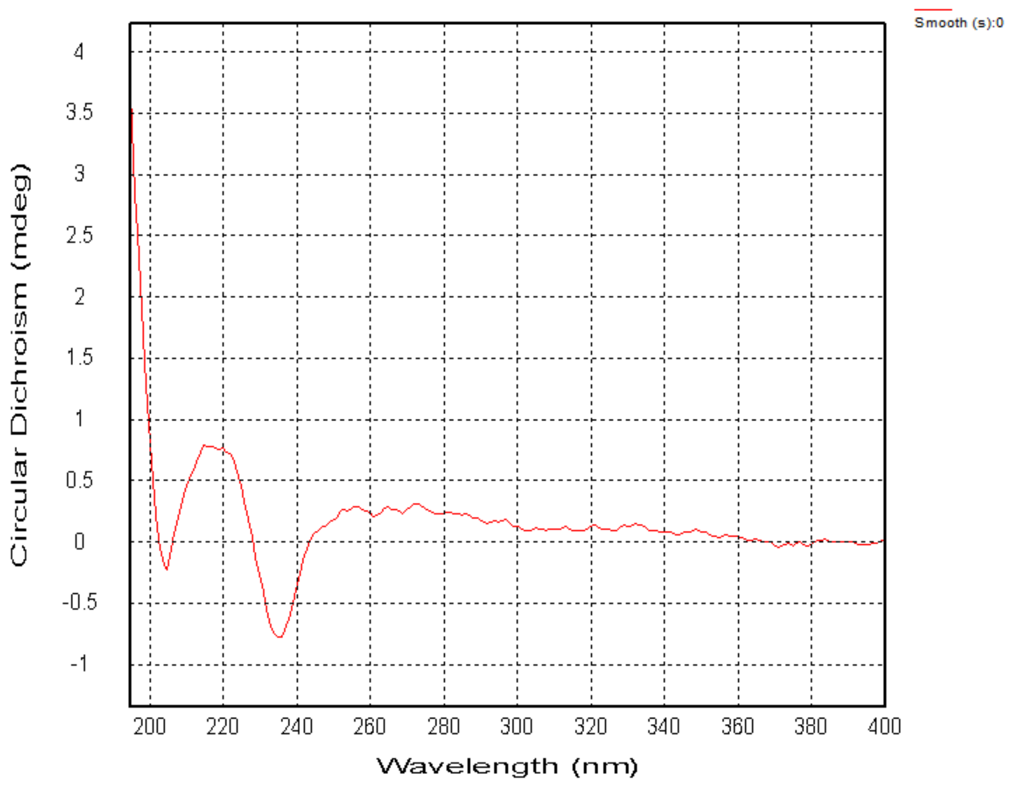

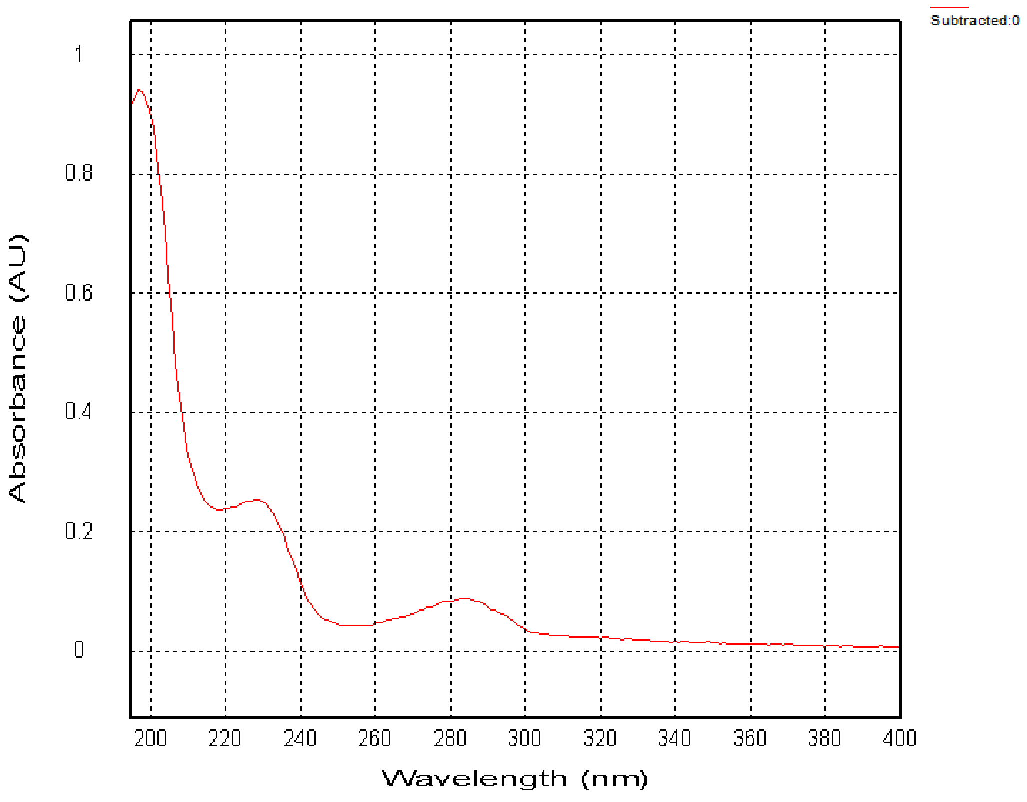

Figure S32. CD and UV spectra of compound **4**

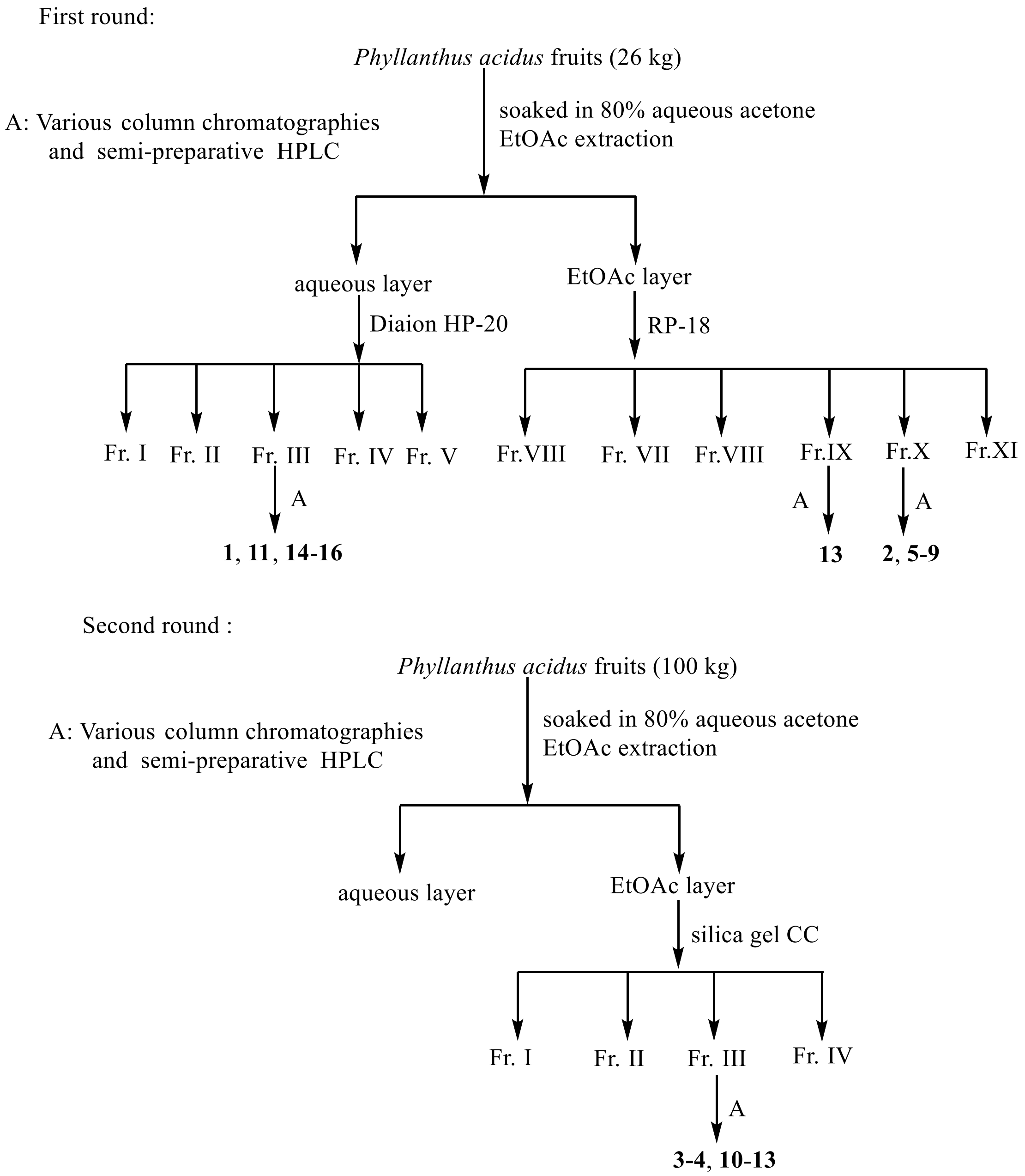

Figure S33. Flowchart of extraction and isolation

Table S1. Inhibitory activities of compounds **3-4**, **10**, **12-15** in ABTS$^+$ inhibition activities assay

**Compd.**	**Concentration（μM）**	**Inhibition rate（%）**	IC_{50} (μM)
Trolox	250	57.26±1.38	176.46±2.07
**3**	250	31.16±0.38	
**4**	250	23.71±0.37	
**10**	250	46.46±0.83	348.43±12.24
**12**	250	49.51±1.60	387.38±8.03
**13**	250	60.12±0.19	203.69±4.66
**14**	250	39.02±1.35	361.75±8.39
**15**	250	49.40±0.72	232.95±1.92

ECD computational details

In general, Conformational analyses were performed through random searching in Sybyl-X 2.0 utilizing the MMFF94S force field, with an energy threshold set at 5 kcal/mol. The findings revealed four lowest energy conformers for compound **3** and two lowest energy conformers for compound **4**. Following that, optimizations of geometry and frequency analyses were conducted at the B3LYP-D3(BJ)/6-31G* level, employing PCM methanol as the solvent, using the ORCA5.0.1 software. All conformers utilized for property calculations in this study were confirmed as stable points on potential energy surface (PES), exhibiting no imaginary frequencies. Using the ORCA5.0.1 software package, the transition energies, oscillator strengths, and rotational strengths (velocity gauge) for the initial 60 excited states were computed with the TD-DFT approach at the PBE0/def2-TZVP level of theory, taking into account the polarizable continuum model (PCM) to simulate methanol solvent effects [1]. The ECD spectra were simulated through overlapping Gaussian functions, where the bandwidth at 1/e peak height is half, with a sigma (σ) value of 0.30 in all cases [2]. Thermal corrections at the B3LYP-D3(BJ)/6-31G* level were applied to ascertain the Gibbs free energies of the conformations, while ORCA5.0 was utilized to assess the electronic energies in PCM methanol at the wB97M-V/def2-TZVP level [1]. To obtain the final spectra, the simulated spectra from the conformers were averaged based on the Boltzmann distribution, taking into account their relative Gibbs free energy (ΔG). The absolute configuration of the sole chiral center was established by aligning the experimental spectra with those predicted for the model molecules.

Table S2. Gibbs free energies[a] and equilibrium populations [b] of low-energy conformers of **3** *R/S*.

Conformers	ΔG (a.u.)	P (%)/100	G (a.u.)
**3***R/S*_opt000001_tddft_	0.00222	4.32	-1265.737145
**3***R/S*_opt000002_tddft_	0.0002	36.47	-1265.73916
**3***R/S*_opt000006_tddft_	0.00111	13.91	-1265.73825
**3***R/S*_opt000007_tddft_	0.00000	45.3	-1265.739364

[a] wB97M-V/def2-TZVP, in a.u. [b] From ΔG values at 298.15K.

Table S3. Cartesian coordinates for the low-energy reoptimized random reseach conformers of **3** *R/S* at B3LYP-D3(BJ)/6-31G* level of theory in methanol.

**3** *R/S* _opt000001_en_		Standard Orientation (A.U.)			
Center number	Atomic number	Atomic Type	X	Y	Z
0	6	0	-9.128231	-5.419195	1.813699
1	6	0	-6.599409	-5.543491	1.107338
2	6	0	-4.826933	-3.875507	2.111451
3	6	0	-5.63609	-2.067882	3.856307
4	6	0	-8.149981	-1.92826	4.585831
5	6	0	-9.903965	-3.606272	3.55961
6	8	0	-12.355986	-3.387925	4.349945
7	6	0	-2.13619	-3.97283	1.200774
8	8	0	-0.396212	-3.416655	3.278873
9	6	0	0.578858	-1.052134	2.874302
10	6	0	-0.051298	-0.051501	0.529192
11	6	0	-1.532192	-2.010074	-0.897723
12	6	0	2.135497	0.28908	4.531606
13	6	0	3.046357	2.628568	3.700884
14	6	0	2.44021	3.641974	1.34463
15	6	0	0.846182	2.272845	-0.264061
16	6	0	0.055849	-3.155811	-3.033988
17	8	0	-1.253121	-5.140631	-4.316458
18	6	0	3.473792	6.204188	0.595264
19	6	0	4.075475	6.490382	-2.225127
20	6	0	5.981684	4.538102	-3.11901
21	8	0	6.463191	4.791185	-5.811883
22	6	0	4.767593	3.68041	-7.361815
23	6	0	5.395977	4.176243	-10.088176
24	8	0	2.957069	2.477947	-6.63765
25	8	0	2.944905	-0.511529	6.845929
26	6	0	1.325286	-2.166813	8.253968
27	1	0	-10.487281	-6.745508	1.024207
28	1	0	-5.996023	-6.966366	-0.248052
29	1	0	-4.278072	-0.758392	4.665558
30	1	0	-8.785088	-0.534194	5.949805
31	1	0	-13.366672	-4.652278	3.514711
32	1	0	-1.690101	-5.886873	0.549858
33	1	0	-3.29073	-1.24354	-1.690211
34	1	0	4.272103	3.658283	4.988766
35	1	0	0.374039	2.965555	-2.137124
36	1	0	0.660047	-1.660679	-4.344577
37	1	0	1.759542	-4.019152	-2.230223

38	1	0	-2.592315	-4.385749	-5.292935
39	1	0	2.111041	7.681174	1.118818
40	1	0	5.182555	6.586596	1.707918
41	1	0	2.34735	6.304528	-3.349537
42	1	0	4.82328	8.390611	-2.57928
43	1	0	7.825984	4.828435	-2.230394
44	1	0	5.321763	2.618331	-2.727928
45	1	0	4.630967	2.657271	-11.252114
46	1	0	4.490295	5.950008	-10.654388
47	1	0	7.428686	4.376613	-10.374889
48	1	0	-0.622626	-1.458449	8.269763
49	1	0	1.343953	-4.086841	7.490369
50	1	0	2.074692	-2.164752	10.17684

**3** *R/S* _opt000002_en_		Standard Orientation (A.U.)			
Center number	Atomic number	Atomic Type	X	Y	Z
0	6	0	-8.631467	-4.881739	0.593926
1	6	0	-6.296531	-5.298762	1.733115
2	6	0	-4.299926	-3.621741	1.386686
3	6	0	-4.686871	-1.479582	-0.11953
4	6	0	-7.00469	-1.032951	-1.249588
5	6	0	-8.9836	-2.742991	-0.899075
6	8	0	-11.228338	-2.208537	-2.06306
7	6	0	-1.765584	-4.140214	2.558078
8	8	0	-0.906632	-1.882782	3.92568
9	6	0	0.975067	-0.805185	2.536879
10	6	0	1.795616	-2.296795	0.53199
11	6	0	0.341967	-4.743338	0.59853
12	6	0	2.024216	1.578049	2.966634
13	6	0	3.925125	2.368871	1.316612
14	6	0	4.738893	0.915182	-0.724185
15	6	0	3.6729	-1.477837	-1.099016
16	6	0	2.0195	-6.966512	1.398151
17	8	0	3.109947	-6.617827	3.824922
18	6	0	6.489994	2.064328	-2.656315
19	6	0	5.19718	2.466642	-5.246731
20	6	0	2.468504	3.334308	-5.071013
21	8	0	2.344791	5.60123	-3.529891
22	6	0	0.299135	5.839012	-2.013555
23	6	0	0.525316	8.103947	-0.315904
24	8	0	-1.470658	4.386762	-1.992908

25	8	0	1.34352	3.192059	4.860982
26	6	0	-1.320531	3.386532	5.38955
27	1	0	-10.175856	-6.207443	0.887875
28	1	0	-6.039362	-6.958284	2.918127
29	1	0	-3.171961	-0.121565	-0.407719
30	1	0	-7.308913	0.635248	-2.403471
31	1	0	-12.43262	-3.516511	-1.66683
32	1	0	-1.908409	-5.642176	3.97541
33	1	0	-0.501753	-5.186334	-1.244196
34	1	0	4.705909	4.245335	1.613315
35	1	0	4.253646	-2.641344	-2.69257
36	1	0	0.883548	-8.69576	1.523297
37	1	0	3.482831	-7.267576	-0.055933
38	1	0	4.022933	-5.035587	3.795471
39	1	0	7.156844	3.889544	-1.948166
40	1	0	8.16958	0.880395	-2.936337
41	1	0	6.31588	3.805822	-6.364999
42	1	0	5.169044	0.684014	-6.308849
43	1	0	1.703095	3.790593	-6.941643
44	1	0	1.273732	1.88891	-4.204687
45	1	0	0.988803	7.41998	1.58408
46	1	0	1.9971	9.404655	-0.942056
47	1	0	-1.300646	9.059846	-0.212447
48	1	0	-2.005344	1.75129	6.449724
49	1	0	-1.532908	5.100624	6.519729
50	1	0	-2.395176	3.569616	3.625654

**3** *R/S* _opt000006_en_		Standard Orientation (A.U.)			
Center number	Atomic number	Atomic Type	X	Y	Z
0	6	0	-8.906531	-7.629638	2.414519
1	6	0	-6.281613	-7.670126	2.58999
2	6	0	-4.865176	-5.479242	2.258632
3	6	0	-6.135841	-3.213349	1.763404
4	6	0	-8.746427	-3.140195	1.597445
5	6	0	-10.139757	-5.358568	1.91799
6	8	0	-12.709098	-5.173988	1.731567
7	6	0	-2.028482	-5.55914	2.372811
8	8	0	-1.105515	-3.641585	4.139209
9	6	0	0.007428	-1.762154	2.743664
10	6	0	0.281606	-2.364827	0.201283
11	6	0	-0.710976	-5.003105	-0.194838

12	6	0	0.859773	0.546372	3.696009
13	6	0	2.031853	2.190608	1.993335
14	6	0	2.346154	1.598194	-0.556488
15	6	0	1.44192	-0.714646	-1.467734
16	6	0	1.387578	-6.912658	-0.722084
17	8	0	2.390459	-6.388953	-3.164422
18	6	0	3.516963	3.490531	-2.33442
19	6	0	1.530256	5.289383	-3.447937
20	6	0	2.600104	7.164876	-5.335355
21	8	0	4.34888	8.880296	-4.093992
22	6	0	6.85123	8.467212	-4.443377
23	6	0	8.386616	10.305114	-2.910943
24	8	0	7.721877	6.822495	-5.769214
25	8	0	0.733075	1.297324	6.163291
26	6	0	-1.448781	0.545921	7.58608
27	1	0	-9.986516	-9.358608	2.683624
28	1	0	-5.331252	-9.446042	2.999012
29	1	0	-5.059067	-1.481997	1.511808
30	1	0	-9.741079	-1.386336	1.221091
31	1	0	-13.448531	-6.818995	1.987799
32	1	0	-1.422943	-7.388126	3.141546
33	1	0	-2.082061	-5.095021	-1.746509
34	1	0	2.698425	3.988609	2.73229
35	1	0	1.674681	-1.231207	-3.43999
36	1	0	2.845019	-6.743185	0.756834
37	1	0	0.590058	-8.839123	-0.64612
38	1	0	3.936392	-7.32411	-3.371103
39	1	0	4.954596	4.595123	-1.330147
40	1	0	4.491027	2.510055	-3.878384
41	1	0	0.070797	4.177507	-4.420098
42	1	0	0.577682	6.308201	-1.912407
43	1	0	3.581938	6.207706	-6.88393
44	1	0	1.108324	8.376629	-6.099529
45	1	0	10.290275	10.422725	-3.689531
46	1	0	7.494329	12.164951	-2.852162
47	1	0	8.507844	9.600745	-0.96766
48	1	0	-1.3425	-1.436026	8.162177
49	1	0	-1.470611	1.763624	9.252685
50	1	0	-3.182906	0.84303	6.488914

**3***R/S*_opt000007_en_		Standard Orientation (A.U.)			
Center	Atomic	Atomic Type	X	Y	Z

number	number				
0	6	0	-8.757727	-2.556316	6.91251
1	6	0	-8.463467	-2.001315	4.35707
2	6	0	-6.079838	-1.948523	3.248029
3	6	0	-3.971134	-2.487646	4.751306
4	6	0	-4.233423	-3.059668	7.290599
5	6	0	-6.638633	-3.08821	8.379077
6	8	0	-6.785753	-3.658328	10.893203
7	6	0	-5.763871	-1.293631	0.507147
8	8	0	-4.43885	-3.369062	-0.779788
9	6	0	-2.154452	-2.483234	-1.587102
10	6	0	-1.811128	0.088886	-1.177031
11	6	0	-4.174238	1.132321	0.020388
12	6	0	-0.279575	-3.925067	-2.770683
13	6	0	1.900353	-2.657093	-3.556941
14	6	0	2.248831	-0.068969	-3.195073
15	6	0	0.35941	1.322296	-1.974598
16	6	0	-5.521527	3.009652	-1.726534
17	8	0	-6.172596	1.92059	-4.091806
18	6	0	4.690237	1.175916	-3.968695
19	6	0	6.634719	1.176449	-1.813617
20	6	0	9.116539	2.427219	-2.523948
21	8	0	8.749137	5.04983	-3.255633
22	6	0	8.628891	6.746519	-1.343839
23	6	0	8.308241	9.383648	-2.355997
24	8	0	8.812381	6.200223	0.867734
25	8	0	-0.422988	-6.438091	-3.31631
26	6	0	-1.860991	-8.022293	-1.646913
27	1	0	-10.634474	-2.590734	7.752046
28	1	0	-10.125462	-1.608623	3.214375
29	1	0	-2.095837	-2.468424	3.909872
30	1	0	-2.60543	-3.48642	8.462959
31	1	0	-8.530032	-3.614098	11.416904
32	1	0	-7.611237	-1.145187	-0.417571
33	1	0	-3.762209	2.090745	1.814859
34	1	0	3.351847	-3.778746	-4.482733
35	1	0	0.598455	3.339652	-1.656029
36	1	0	-7.303543	3.621507	-0.863177
37	1	0	-4.320124	4.694413	-1.971088
38	1	0	-4.632145	1.286739	-4.842744
39	1	0	5.491175	0.197143	-5.613369
40	1	0	4.326143	3.130515	-4.551644
41	1	0	5.845145	2.116138	-0.144617

42	1	0	7.043716	-0.780435	-1.254401
43	1	0	10.46254	2.349144	-0.955577
44	1	0	9.946844	1.547418	-4.202487
45	1	0	7.085426	9.407045	-4.018577
46	1	0	7.559311	10.600924	-0.872253
47	1	0	10.164027	10.100614	-2.928522
48	1	0	-1.280283	-9.952858	-2.087905
49	1	0	-1.422081	-7.598426	0.333384
50	1	0	-3.892433	-7.806845	-1.955713

Table S4. Gibbs free energies[a] and equilibrium populations[b] of low-energy conformers of **4** *R*/*S*.

Conformers	ΔG (a.u.)	P (%)/100	G (a.u.)
**4***R/S*_opt000001_tddft_	0.0	88.28	-1151.243617
**4***R/S*_opt000002_tddft_	0.00191	11.71	-1151.24171

[a] wB97M-V/def2-TZVP, in a.u. [b] From ΔG values at 298.15K.

Table S5. Cartesian coordinates for the low-energy reoptimized random reseach conformers of **4** *R/S* at B3LYP-D3(BJ)/6-31G* level of theory in methanol.

**4** *R/S* _opt000001_en_		Standard Orientation (A.U.)			
Center number	Atomic number	Atomic Type	X	Y	Z
0	6	0	-5.670282	-5.40076	4.245889
1	6	0	-3.899868	-3.644522	5.061727
2	6	0	-3.704825	-1.279974	3.907787
3	6	0	-5.334027	-0.702018	1.92197
4	6	0	-7.1069	-2.447801	1.079968
5	6	0	-7.276267	-4.801879	2.240819
6	8	0	-9.055646	-6.450067	1.342992
7	6	0	-1.706291	0.536388	4.83143
8	8	0	-1.964162	2.977521	3.585638
9	6	0	-0.139766	3.099559	1.762027
10	6	0	1.654748	1.19061	1.960915
11	6	0	1.02554	-0.383481	4.239851
12	6	0	-0.022041	4.916189	-0.121013
13	6	0	1.984457	4.762177	-1.823988
14	6	0	3.819844	2.864262	-1.678754
15	6	0	3.634778	1.065733	0.252646
16	6	0	2.843366	0.126463	6.413139
17	8	0	2.077652	-1.434697	8.483454
18	6	0	5.975796	2.794385	-3.553877
19	6	0	5.187188	2.403584	-6.32393
20	6	0	4.425611	-0.287514	-6.984386

21	8	0	2.431078	-1.257141	-5.383099
22	6	0	0.075314	-0.362281	-5.850332
23	6	0	-1.760065	-1.314264	-3.912193
24	8	0	-0.424996	1.040007	-7.586085
25	1	0	-5.805433	-7.233818	5.168661
26	1	0	-2.645764	-4.118064	6.621502
27	1	0	-5.22218	1.139852	1.028407
28	1	0	-8.378016	-2.004741	-0.467967
29	1	0	-8.983646	-7.991023	2.311244
30	1	0	-1.941047	0.876707	6.860935
31	1	0	1.02726	-2.416468	3.839168
32	1	0	-1.439116	6.391129	-0.263086
33	1	0	2.110131	6.165363	-3.320398
34	1	0	5.029029	-0.440311	0.381272
35	1	0	4.783666	-0.322323	5.808743
36	1	0	2.769319	2.146644	6.915919
37	1	0	3.083431	-1.004193	9.936805
38	1	0	7.012385	4.588109	-3.431062
39	1	0	7.315218	1.30995	-3.001093
40	1	0	3.649399	3.694379	-6.825276
41	1	0	6.788924	2.879302	-7.55639
42	1	0	3.825889	-0.403808	-8.962847
43	1	0	5.998468	-1.593402	-6.663626
44	1	0	-1.430488	-0.29071	-2.142608
45	1	0	-3.687246	-0.966099	-4.549652
46	1	0	-1.4703	-3.3209	-3.52

**4***R/S*_opt000002_en_		Standard Orientation (A.U.)			
Center number	Atomic number	Atomic Type	X	Y	Z
0	6	0	-7.559763	-3.406621	6.445679
1	6	0	-6.272401	-1.441306	5.288821
2	6	0	-5.979384	-1.354685	2.664473
3	6	0	-7.005716	-3.289006	1.211833
4	6	0	-8.296144	-5.279009	2.351178
5	6	0	-8.575862	-5.338825	4.966755
6	8	0	-9.829333	-7.237623	6.196605
7	6	0	-4.510537	0.805318	1.515783
8	8	0	-4.750394	0.802085	-1.224273
9	6	0	-2.545663	-0.144976	-2.183313
10	6	0	-0.652668	-0.382521	-0.373418
11	6	0	-1.629657	0.661189	2.079017
12	6	0	-2.152812	-0.773379	-4.690776

13	6	0	0.247278	-1.657241	-5.34771
14	6	0	2.185019	-1.926512	-3.576482
15	6	0	1.707041	-1.27223	-1.051669
16	6	0	-0.44436	3.251072	2.631432
17	8	0	-1.307118	4.283679	4.973871
18	6	0	4.775431	-2.838777	-4.368567
19	6	0	6.924391	-1.080871	-3.517985
20	6	0	6.556039	1.590819	-4.501856
21	8	0	8.495992	3.274631	-3.536941
22	6	0	8.099698	4.198335	-1.186343
23	6	0	10.284014	5.808179	-0.343182
24	8	0	6.243388	3.745514	0.073787
25	1	0	-7.794785	-3.475389	8.481997
26	1	0	-5.464588	0.053898	6.448683
27	1	0	-6.806374	-3.241468	-0.827815
28	1	0	-9.088885	-6.782987	1.193695
29	1	0	-10.430392	-8.443142	4.970579
30	1	0	-5.322366	2.605062	2.157827
31	1	0	-1.275931	-0.582898	3.69605
32	1	0	-3.642658	-0.571897	-6.085103
33	1	0	0.615153	-2.150872	-7.308725
34	1	0	3.19097	-1.419421	0.362065
35	1	0	1.60825	3.04265	2.789575
36	1	0	-0.813472	4.544024	1.043659
37	1	0	-2.882114	5.145427	4.685207
38	1	0	5.131751	-4.730509	-3.592456
39	1	0	4.81874	-3.040899	-6.432225
40	1	0	7.00728	-1.016985	-1.450336
41	1	0	8.743069	-1.823322	-4.179681
42	1	0	6.764436	1.673939	-6.55791
43	1	0	4.697182	2.322932	-3.967134
44	1	0	9.634916	7.144985	1.084499
45	1	0	11.712085	4.578388	0.514402
46	1	0	11.160179	6.78925	-1.932336

References

1. Neese, F.; The ORCA program system. *WIREs. Comput. Mol. Sci.* **2011**, *2*, 73-78.
2. Stephens, P. J.; Harada, N., ECD cotton effect approximated by the Gaussian curve and other methods. *Chirality* **2009**, *22*, 229-233.
